# Enhanced formation of tertiary lymphoid structures shapes the anti-tumor microenvironment in gastrointestinal stromal tumors after imatinib targeted therapy

**DOI:** 10.7150/thno.123923

**Published:** 2026-01-01

**Authors:** Ping Tao, Jiongyuan Wang, Peidang Fan, Wenshuai Liu, Weiqi Lu, Yujie Hu, Qun Lu, Lijie Ma, Hanxing Tong, Yong Zhang

**Affiliations:** 1 Department of Laboratory Medicine, Shanghai Traditional Chinese Medicine-Integrated Hospital, Shanghai University of Traditional Chinese Medicine, Shanghai, China.; 2 Department of General Surgery, Zhongshan Hospital, Fudan University, Shanghai, China.; 3 Department of General Surgery, Shanghai Xuhui Central Hospital, Shanghai, China.; 4 Department of General Surgery, Xiamen Hospital, Zhongshan Hospital, Fudan University, Xiamen, China; Xiamen Clinical Research Centre for Cancer Therapy, Xiamen, China; Department of General Surgery, Zhongshan Hospital, Fudan University, Shanghai, China.

**Keywords:** GIST, single-cell RNA sequencing, TLS, imatinib, GIST immune classes

## Abstract

**Rationale:** Tertiary lymphoid structures (TLSs) play a key role in the adaptive immune response within the local tumor immune microenvironment (TIME) and can predict the clinical outcome of several solid tumors. However, the clinical relevance of TLSs and their formation mechanism in gastrointestinal stromal tumors (GISTs) remain unclear.

**Methods:** Integration analysis was performed on a single-cell RNA sequencing (scRNA-seq) cohort (n = 8), a public transcriptome cohort (n = 65), a public scRNA-seq cohort (n = 7), a tissue cohort (n = 197) and a serum cohort (n = 169) to decode the characteristics of the immunological microenvironment of GIST. The multiplex immunohistochemistry (mIHC) staining, *in vitro* cell culture, chemotaxis assays and antibody-dependent cellular phagocytosis (ADCP) experiments were used to validate the results of the bioinformatics analysis.

**Results:** Preoperative imatinib targeted therapy significantly enhanced TLS formation in GIST tissues, predicting improved therapeutic efficacy and favorable prognosis. Mechanistically, imatinib remodeled the local microenvironment via tumour-associated macrophages, recruiting B cells via the CXCR4-CXCL12 axis to drive TLS development. Functionally, TLS dominated germinal centre (GC) B-cell differentiation and the formation of IgG^+^ plasma cells (PCs), which preferentially enhanced the adaptive immune response through the ADCP effect. From a clinical perspective, we identified three distinct GIST immune classes (GICs A-C). GIC-A tumors featured abundant CD20^+^ B cells and TLS, as well as a favorable prognosis. They were accompanied by enhanced antigen presentation, accumulation of IgG^+^ PCs, increased immunosuppressive properties and a high frequency of *KIT* exon 11 mutations. These mutations potentially serve as a predictive biomarker for future targeted and immunotherapies. Furthermore, patients with high serum IgG levels experienced significant therapeutic benefits.

**Conclusions:** Our data show that local adaptive immunity dominated by TLS is a key factor in the efficacy of targeted therapy, and suggest that inducing IgG could be a feasible strategy for improving the prognosis of patients with GIST.

## Introduction

Gastrointestinal stromal tumor (GIST) is defined as the most common soft tissue sarcoma (STS) of the gastrointestinal tract and featured with activating mutations in *KIT* or, less commonly, *PDGFRA* 1. Exploring the molecular subtypes of GIST at the time of diagnosis has played a critical role in clinical decision-making, particularly in adjuvant or metastatic settings, since the distinct molecular biology and microenvironmental features of GIST affect treatment response and clinical outcomes 2,3. However, mutations in type III receptor tyrosine kinases (RTKs) account for over 85% of GISTs, and most primary *KIT* mutations respond to treatment with the tyrosine kinase inhibitor (TKI), imatinib 4. Notably, imatinib-resistance develops most commonly due to the widely secondary kinase mutations 5. As reported, only a limited number of immunotherapy trials have been performed for GIST, such as those involving anti-CTLA-4 or anti-PD-1 drugs, and the available clinical data are not very promising 6-9, due to the heterogeneous and unresolved tumor microenvironment (TIME). Therefore, it is necessary to characterize the TIME, which comprises various cellular components that play a crucial role in the progression of GIST and its response to imatinib.

GIST is characterized as an immune-infiltrated yet immunosuppressive tumor, primarily due to modulation of the TIME 10. Single-cell atlas of primary GIST has revealed two types of CD8^+^ effector memory T-cell subset with the highest clonal expansion, which exhibit cytotoxicity but an exhausted phenotype 11. Furthermore, imatinib decreases the density of effector CD8^+^ T cells, while increasing the naive CD8^+^ T cell subset in the murine GIST model. This is consistent with changes in chemokine production and CD8^+^ T cell recruitment. However, imatinib was unable to induce intra-tumoral T cell receptor (TCR) clonal expansion 12. Furthermore, an increased number of Treg cells, but a decreased number of CD8^+^ T cells and plasma cells (PCs), were found in the TLS with an imatinib-resistant phenotype 13. Together, previous single-cell analyses of GIST have largely focused on malignant cells or different T-cell subsets, but limited attention to B-cell infiltration of the intra-tumoral area and the influence of B cells on the imatinib response of GIST. Thus, a high-resolution immune landscape of the tumor is urgently needed to create a comprehensive B-cell atlas in GISTs that respond differently to imatinib therapy.

Here, we analyzed the GIST TIME landscape using single-cell RNA sequencing (scRNA-seq), comparing the unique cellular profiles of eight tumor and paired non-tumor tissues. We also demonstrated that GIST patients may benefit from imatinib-targeted therapy due to TIME remodeling by tumor-infiltrating B cells in TLS, which were recruited by tumour-associated macrophages via the CXCR4-CXCL12 axis. Functionally, TLS dominated GC-B cell differentiation and IgG^+^ PCs enhanced the adaptive immune response preferentially through antibody-dependent cellular phagocytosis (ADCP) effect. From a clinical perspective, three distinct GIST immune classes (GICs A-C) were identified. GIC-A was characterized by the presence of CD20^+^ B cells in TLS, strong antigen presentation, accumulation of IgG PCs, immunosuppressive properties, and a high frequency of *KIT* exon 11 mutations, which predicted favorable outcomes, and potentially served as a predictive biomarker for future targeted and immunotherapies. Furthermore, patients with high serum IgG levels were shown to experience significant therapeutic benefits. Taken together, our results provide deep insight into B cell and TLS functions within the TIME and could inform novel therapeutic strategies for GIST.

## Materials and Methods

### Patients and GIST samples

For the pathology evaluation, 197 GIST patients who underwent radical surgery at Zhongshan Hospital (Fudan University, China) between 2011 and 2021 were enrolled in the present retrospective study. For scRNA-seq, fresh surgical GIST tumor specimens and matched peri-tumor specimens (n = 8) were selected for further analysis. All tumor and peri-tumor GIST samples were confirmed by two pathologists post-surgery. The inclusion criteria were described as follows: (1) a preoperative needle biopsy or histopathology indicated GIST; (2) no preoperative medication was administered, or treatment was limited to imatinib alone; (3) there was no evidence of distant metastasis; (4) the patient had no prior malignancies or concurrent severe medical conditions; and (5) complete pathological and follow-up data were available. The exclusion criteria were as follows: (1) inconsistent results between preoperative biopsy and postoperative pathology; (2) distant metastasis; and (3) missing follow-up and clinical data. Serum samples from 169 GIST patients who underwent radical surgery at Zhongshan Hospital (Fudan University, China) between 2013 and 2019 were retrospectively enrolled in the present study for serum IgG (cutoff value: 1229538.4 ng/ml) and IgA (cutoff value: 31.629035 ng/ml) detection. The demographics and tumor clinicopathological features of all GIST cohorts are summarized in **Tables S1-S3**.

### Ethical approval and consent

This study was reviewed and approved by the Institutional Review Board of Zhongshan Hospital, Fudan University, China (ID: B2022-586R). Written informed consent was obtained for all participants. All studies were performed in accordance with the Declaration of Helsinki.

### Data acquisition and functional analysis

Public bulk RNA-seq TPM matrix and scRNA-seq data of Gene Expression Omnibus (GEO) datasets (GSE136755, GSE35998, and GSE254762) were downloaded from www.ncbi.nlm.nih.gov/geo/ and re-analyzed in the present study. Additionally, all the public datasets, TPM (transcripts per kilobase of exon model per million mapped reads) values, were normalized using the log_2_ (TPM + 1) transformation. The differentially expressed genes (DEGs) between the groups were visualised using the R package “Limma”. Gene Ontology (GO) and Kyoto Encyclopedia of Genes and Genomes (KEGG) analyses were performed using the “clusterProfiler” R package and visualised using Metascape5 14. A gene dataset based on “GO biological process” was downloaded from the Molecular Signature Database and Gene Set Enrichment Analysis (GSEA) was conducted to analyse the differences between subtypes.

### Gene signatures analysis

The gene signatures used to determine the functional orientation are described as follows 15. Enrichment scores were calculated using the single-sample gene set enrichment analysis (ssGSEA) method implemented in the R package. Each signature is summarized in **Table S4**.

### Single-cell RNA sequencing

The single-cell suspensions were converted into barcoded scRNA-seq libraries using the Chromium Single Cell Library, Gel Bead and Chip Kits (10x Genomics), with the aim of achieving 6,000 cells per library. Samples were processed using V2 barcoding chemistry kits from 10x Genomics. Single samples were always processed in a single well of a PCR plate to allow all cells from a sample to be treated with the same master mix in the same reaction vessel. For each experiment, all samples were processed in parallel in the same thermal cycler. The libraries were sequenced on an Illumina HiSeq 4000 and mapped to either the human genome (build GRCh38) or the mouse genome (build mm10) using CellRanger software (version 3.0.2, 10x Genomics).

### Single-cell transcriptome data processing

The cell-gene count matrix output was processed for quality control and downstream analysis using the Seurat (version 3.1.0) package in R (version 3.6.1). Cells with fewer than 200 genes or more than 40% mitochondrial genes were removed from the analysis as they were considered low quality. Since cells from tumour and adjacent normal tissues were loaded in batches for each patient, data for each patient was generated as an individual Seurat object. These Seurat objects were then integrated using the Harmony algorithm (Harmony R package, version 1.0). The top 50 principal components (PCAs) were used for graph-based clustering to identify distinct cell groups at the indicated resolution. For the subgroup analysis, graph-based clustering of each cell cluster was performed using the significant PCAs identified by the ElbowPlot() function, in order to identify subgroup cells based on t-SNE analysis 16. The cell types were defined based on the expression of their respective canonical marker genes (**Table S4**).

### Multiplexed immunohistochemistry

Seven-color staining with different panels was performed using the PHENOIMAGER™ platform, which incorporates the PhenoImager HT quantitative pathology imaging system and inForm image analysis software from Akoya Bioscience's Phenoptics Research Solution 17. The slides were first deparaffinized and rehydrated, followed by microwave antigen retrieval at pH 9.0. After blocking the endogenous peroxidase and non-specific binding sites, the primary antibodies and secondary HRP-conjugated polymers were applied. Each HRP-conjugated polymer covalently binds a distinct fluorophore via tyramide signal amplification. This covalent reaction was followed by an additional antigen retrieval step (pH = 6.0) to remove the background signal before the next step. The following fluorescent dyes were then applied in order: Opal-620, Opal-690, Opal-480, Opal-570, Opal-520 and Opal-780. After six sequential reactions, the slides were counterstained with DAPI and mounted with fluorescence mounting medium. The antibodies used for mIHC staining are listed in **Table S5**. The slides were scanned using the Vectra 3 automated, high-throughput, multiplexed biomarker imaging system (Akoya Phenomics HT).

### Immune cell isolation and chemotaxis assays

Splenic B cells were isolated from mice using respective isolation kits (purity above 90%; STEMCELL Technologies, Vancouver, Canada). C57BL/6J mice were acclimated to a 12 h light-dark cycle for at least five days prior to conducting experiments involving the isolation and culture of mouse macrophages were conducted. Subsequently, peritoneal cavity macrophages (PCDMs) were isolated. To stimulate a substantial yield of macrophages, 1 ml of sterile 3% thioglycolic acid broth was administered intraperitoneally daily for 3 days. The mice were then humanely euthanized via cervical dislocation. A 1 ml aliquot of pre-chilled PBS was then injected into the peritoneal cavity and a gentle abdominal massage performed for 3-5 min. The abdominal cavity was carefully opened to retrieve the peritoneal fluid, which was then centrifuged at 500 g and 4 °C for 5 min. The supernatant was discarded.

Subsequently, 1,000 μL of PCDMs (5 x 10⁵) were added to the bottom wells, with 300 μL of B cells (1 x 10⁵) placed in the upper wells. Both the B cells and the PCDMs were cultured in RPMI 1640 supplemented with 10% fetal bovine serum. B cell activation was performed as previously described 18. We stimulated the cells with LPS (10 μg/ml, Sigma-Aldrich) and β-mercaptoethanol (50 nM, Sigma-Aldrich) for three days. The bottom wells contained 20 μl of phosphate-buffered saline, either with or without anti-mouse CXCL12 (50 μg/ml, R&D Systems). The cells were then allowed to migrate for 5 h at 37 °C in an atmosphere containing 5 % CO₂. The cells in the bottom wells were harvested and analysed using flow cytometry. The chemotactic index was calculated as the ratio of migrated B cell numbers to total macrophage numbers.

### *In vitro* ADCP assay

To perform a reproducible *in vitro* model for quantifying ADCP effect of GIST-T1 cells by THP-1 macrophages, mimicking the efferocytosis defect observed in GIST microenvironment, which coupling imatinib induced apoptosis with Fcγ-receptor-mediated engulfment by THP-1 macrophages. GIST-T1 cells were plated at 3x10⁴ cells/cm in complete medium. After 4 h adherence, switched to condition medium containing imatinib (50 µM, MCE) for 24 h. Replacing medium with 50 µM imatinib for 24 h (late apoptotic/necrotic), and validated apoptosis of GIST-T1 cells by Annexin V-FITC/PI dual staining (> 70 % Annexin V+/PI-). Then harvested apoptotic GIST-T1 cells, washed twice with PBS, and resuspend at 1x10⁶ cells/mL in PBS with 1 % BSA. Cultured 5x10⁶ THP-1 macrophages per 10 cm Petri dish in medium for 7 days, refreshing on day 3 and 5. Labeled GIST-T1 cells with 2 µM CypHer5E for 30 min at 37 °C, wash twice. Labeled THP-1 macrophages with 2 µM Calcein AM for 30 min, wash twice. Resuspended both populations in complete DMEM/F-12 without phenol red. Seed THP-1 macrophages at 1x10⁵ cells per well in 96-well glass-bottom plate; allow 2 h adherence. Add apoptotic GIST-T1 cells at target effector ratio = 1 : 2 (5 x 10⁴ GIST-T1 cells per well) with 100 µl human IVIG (5mg/ml, Boyar Biotech). Without imatinib treatment was performed as control (spontaneous phagocytosis), and FcR receptor blockade (anti-CD16, Biolegend) was defined as experimental group. Centrifuged plate 30 s at 200 g to synchronize contact and incubated with 37 °C, 5% CO₂ for 90 min (kinetic optimization range 30-180 min). Gently washed wells 3xwith warm PBS to remove non-ingested targets. Fix with 4 % PFA 15 min RT. Counter stained nuclei with DAPI (1 µg/mL, 5 min). Acquired ≥ 5,000 THP-1 macrophages events per well and gated single cells by flow cytometry.

### Flow cytometry

Mononuclear cells were stained with 7-AAD in order to filter out dead cells. For surface phenotype staining, the cells were incubated with antibodies in MACS buffer at room temperature for 15 min. Intracellular antibodies were then stained in permeabilization buffer for 30 min at 4 °C (FITC anti-mouse CD19 antibody (1D3/CD19, BioLegend); APC anti-mouse CD68 antibody (FA-11, BioLegend)). Data were acquired using an LSR Fortessa flow cytometer (BD Biosciences) and analysed using FlowJo software (version 10.8.1, BD Biosciences).

### Enzyme linked immunosorbent assay

The levels of IgG and IgA in serum were quantified using IgG and IgA ELISA kits according to the manufacturers' protocols. Briefly, flat-bottomed 96-well plates were pre-coated with either an IgG antibody or an IgA capture antibody. The plates were then incubated with diluted serum samples in assay buffer for two hours at room temperature. After six washes with washing buffer, human IgG and IgA antibodies (2 mg/ml) were added and the plates were incubated for one hour at room temperature. Following a further six washes, the plates were developed by adding the TMB substrate, after which the absorbance was read at 450/570 nm using a VICTOR Nivo Multimode Microplate Reader (PerkinElmer).

### Statistical analysis

All statistical analyses were performed using SPSS 22.0 (SPSS Inc., Chicago, IL, USA) and R 4.0.4 (R Foundation for Statistical Computing, Vienna, Austria; http://www.r-project.org/). Kaplan-Meier survival analyses were performed using the R software and the corresponding R packages. Continuous data are expressed as the mean ± standard error of the mean (SEM). An unpaired t-test or Mann-Whitney test was applied as appropriate for the data analysis. Spearman's correlation was used to compute correlations. Adjusted p-values were calculated using the Benjamini-Hochberg method to control the false discovery rate. The significance levels of the tests performed are denoted by asterisks: ****P < 0.0001, ***P < 0.001, **P < 0.01 and *P < 0.05.

## Results

### High-resolution landscape of the tumoral ecosystem in GIST by single cell profiling

**Figure 1:**
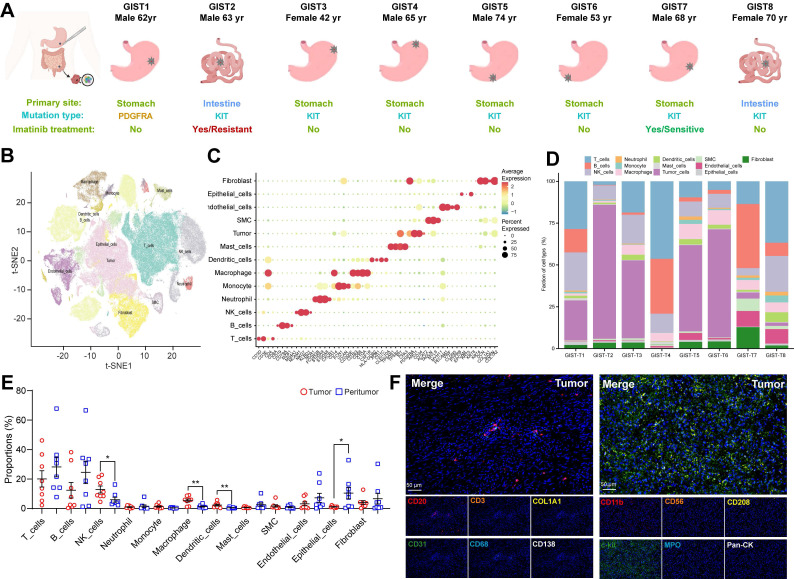
** High-resolution landscape of the tumoral ecosystem in GIST by single cell profiling. (A)** Study overview. Resected tumor tissues were digested to single-cell suspensions, and subjected to single-cell assays shown. Clinical data of patients indicating summary primary site, mutation type, and Imatinib treatment. **(B)** t-Distributed stochastic neighbour embedding (t-SNE) plot of all immune scRNA-seq clusters, with each color representing one cluster. **(C)** Expression of cell-type marker genes across immune scRNA-seq clusters. Heatmap shows the average expression per cell. Clusters are shown using even sampling of cells from eight patients. **(D)** Proportions of the immune scRNA-seq clusters in GIST tumor tissue in individual samples (n = 8). **(E)** Dot plots showing the proportions of immune scRNA-seq clusters from peri-tumor (n = 8) and tumor (n = 8) tissues. **(F)** Representative mIHC staining of CD3^+^ T cells, CD20^+^ B cells, CD31^+^ endothelial cells, CD68^+^ macrophages, CD138^+^ PCs, COL1A1^+^ fibroblasts, CD11b^+^ myeloid cells, CD56^+^ NK cells, CD208^+^ dendritic cells, c-kit^+^ mast cells, MPO^+^ neutrophils, and Pan-CK^+^ epithelial cells in GIST tumor tissues. Scale bars, 50 μm. All data were displayed as mean ± SEM. * *P* < 0.05.

To explore the complexity of cellular profiling in GIST, we performed scRNA-seq analysis with unsorted cells from surgical tumor specimens, including intra-tumor, and matched peri-tumor tissues from eight GIST patients (**Figure 1A, S1A**). Two GIST patients had previously received targeted therapy with imatinib: one had developed imatinib resistance, while the other was imatinib sensitive. In addition, a patient with a locally advanced GIST and a *PDGFRA* exon 18 D842V mutation was also enrolled in the present study. Detailed information, including tumor stage, tumor size, treatment and gene mutations, were provided in **Table S2**.

To ensure that all cells were of high quality and devoid of potential contaminants, we performed quality control, doublet removal, multiple cell-type signature visualization, and batch correction. The nFeature_RNA, nCount_RNA, and percent.mt were evaluated (**Figure S1B**). In total, we cataloged 153515 high-quality single cells into 13 major cell lineages annotated by canonical marker expression and visualized using t-Distributed Stochastic Neighbor Embedding and projection (tSNE) (**Figure 1B**). Different cell lineages were annotated with typical cell markers as follows: GIST tumor cells (KIT^+^ and PDGFRA^+^), epithelial cells (EPCAM^+^), T cells (CD3D^+^), NK (natural killer) cells (KLRB1^+^), B cells (CD79A^+^), plasma cells (CD38^+^), macrophages (CD68^+^), dendritic cells (CD14^+^), mast cells (TPSAB1^+^), neutrophils (S100A9^+^), endothelial cells (PECAM1^+^), fibroblasts (COL1A1^+^), and smooth muscle cells (SMC) (ACAT2^+^) (**Figure 1C, S1C**).

Consistent with previous findings in other solid tumors 19, it is noteworthy that all of these cell subtypes were shared in all eight GIST patients, with the difference being the infiltration rate of each cell type. The proportion of malignant and normal clusters varied significantly, suggesting considerable intertumoral heterogeneity and the formation of patient-specific clusters (**Figure 1D, S1D**). Meanwhile, tumor-infiltrating immune cells were found to exhibit significant heterogeneity within eight GIST patients, as well as between paired tumor and peri-tumor GISTs. Notably, NK cells, macrophages, and dendritic cells were found to be significantly more prevalent in tumor tissue, whereas epithelial cells, B cells, and T cells were predominantly present in adjacent peri-tumor tissues (**Figure 1E**). The heterogeneous feature of TIME in GIST was further verified and visualized *in situ* by multiplex immunohistochemistry (mIHC) through two immune panels (**Figure 1F, S1E-F**). Similar results were also validated in the public scRNA-seq dataset (GSE254762), including surgical tumor specimens from seven GIST patients, with four specimens from three patients exhibiting imatinib resistance and five specimens from four patients exhibiting imatinib sensitive (**Figure S1G-H, Table S6**).

In summary, the integrated scRNA-seq analysis demonstrated that the GIST TIME harbored a complex and heterogeneous ecosystem with various immune cell infiltration.

### Spatial infiltration features of B cell subsets in GIST with imatinib targeted therapy

**Figure 2:**
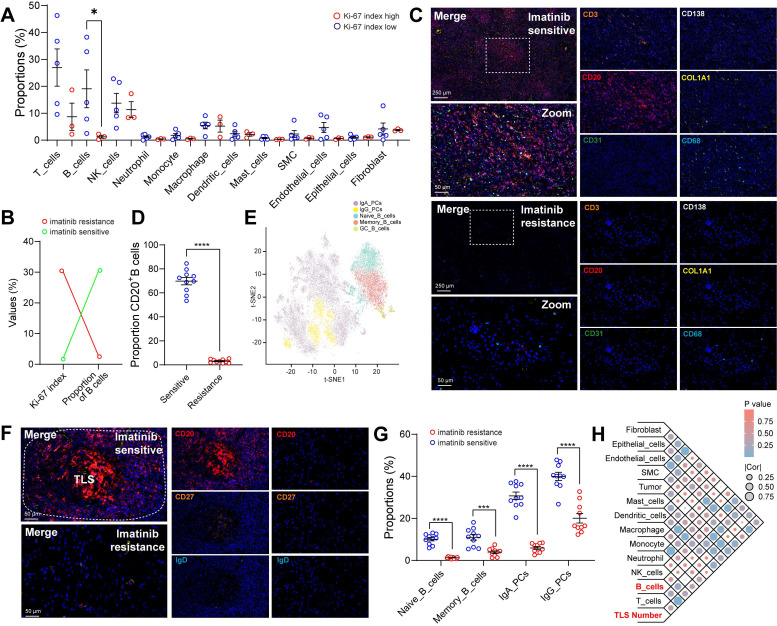
** Spatial infiltration features of B cell subsets in GIST with Imatinib targeted therapy. (A)** Dot plots showing the comparison of the proportions of immune scRNA-seq clusters between Ki-67 low (n = 5) and high (n = 3) groups (Mann-Whitney test). **(B)** Dot plots showing the Ki-67 index and proportion of B cells between Imatinib resistance and sensitive GIST. **(C)** Representative mIHC staining of CD3^+^ T cells, CD20^+^ B cells, CD31^+^ endothelial cells, CD68^+^ macrophages, CD138^+^ PCs, and COL1A1^+^ fibroblasts in tumor tissues between Imatinib resistance and sensitive GIST. Scale bars, 50 μm. **(D)** Dot plots showing the comparison of CD20^+^ B cells in tumor tissues between Imatinib resistance (n = 10) and sensitive (n = 10) GIST (Mann-Whitney test). **(E)** t-SNE plot of all B cells, with each colour representing one of the five subgroups. **(F)** Representative mIHC staining of CD20^+^ B cells, IGHD^+^ Naïve and CD27^+^ Memory B cells between Imatinib resistance (n = 10) and sensitive (n = 10) GIST. Scale bars, 50 μm. **(G)** Dot plots showing the comparison of CD20^+^ B cells, IGHD^+^ Naïve B cells, CD27^+^ Memory B cells, IgA^+^ PCs, and IgG^+^ PCs between Imatinib resistance (n = 10) and sensitive (n = 10) GIST (Mann-Whitney test). **(H)** Bubble diagram depicting the spearman correlation between TLS number and immune scRNA-seq clusters in GIST scRNA-seq cohort (n = 8). All data were displayed as mean ± SEM. * *P* < 0.05; *** *P* < 0.001; **** *P* < 0.0001.

To investigate the correlation between clinical characteristics and cell composition in the TIME of GIST, these eight cases were divided based on distinct clinical features (tissue source, gender, mutation site, tumor source, tumour status, tumour treatment, National Institutes of Health (NIH) grading and Ki-67 index (**Figure S2A, Table S2**). Remarkably, we found that patients with a low Ki-67 index (≤ 5%) had significantly more B-cell infiltration than those with a high Ki-67 index (> 5%), but no other immune subsets were affected (**Figure 2A**). Furthermore, the Ki-67 index was found to be negatively correlated with the proportion of B cells (**Figure S2B**). Notably, preoperative imatinib resistance GISTs presented the highest Ki-67 index (30%), but the lowest proportion of B cells (0.28%). In contrast, preoperative imatinib sensitive GISTs presented the lowest Ki-67 index (2%), but the highest proportion of B cells (38.28%) (**Figure 2B**). Meanwhile, the public scRNA-seq cohort validated that the mitotic index was > 5 in three of the four imatinib resistance cases and < 5 in all five imatinib sensitive cases. Meanwhile, gene mutations in most of the imatinib sensitive cases were *KIT* exon 11 (**Figure S2C**). Meanwhile, limited B cells, but enriched fibroblasts were observed in the primary GIST tumor site within the imatinib resistance cases (MP1, MP3, and ML1), compared with those in the imatinib sensitive case (PG4) (**Figure S1H**). In line with the scRNA-seq data, we further validated the spatial distribution features of B cells, with significant more infiltration of CD20^+^ B cells in the imatinib sensitive GIST, compared with those in the imatinib resistance GIST tumor tissue (**Figure 2C-D**) and peri-tumor tissues (**Figure S2D-E**).

In order to decode the transcriptional atlas of B cells in GIST, we re-examined our scRNA-seq profiles of 23,654 B cells and identified five distinct clusters of B cells: Naive_B_cells, Memory_B_cells, GC_B_cells, IgA_PCs and IgG_PCs (**Figure 2E**), through publicly available gene expression profiles 20. Additionally, Naive_B_cells expressed distinct signature genes, such as *IGHD*, *FCER2*, *TCL1A* and *IL4R*, whereas Memory_B_cells expressed *CD27*, *AIM2* and *TNFRSF13B*. GC_B_cells were featured with the specific expression of *LRMP*, *SUGCT*, and *MME*. The presence of *MKI67* and *AICDA* in GC_B_cells further highlights their ability to proliferate and undergo somatic hypermutation (**Figure S2F**).

However, only 22 B cells were discovered in imatinib resistance GIST (GIST-02). In contrast, imatinib sensitive GIST harbored the largest number of 4,222 B cells of all the samples (**Figure 1D, Table S7-8**). Moreover, the mIHC staining further validated that there were abundant CD20^+^ B cells containing IGHD^+^ naïve and CD27^+^ memory B cells, which were mainly localized within the core of TLS in imatinib sensitive GIST, compared with imatinib resistance GIST (**Figure 2F-G**). Meanwhile, the public scRNA-seq cohort validated that imatinib sensitive GIST (PG4) showed the highest proportion of switched memory B cells, while imatinib resistance cases (MP1, MP3, and ML1) indicated high proportions of transitional B cells (**Figure S2H-I**).

As in previous studies, TLS was defined as an effective prognostic factor for overall survival (OS) and was significantly associated with lower imatinib resistance in GIST 21, we also found that the TLS number was significantly and positively correlated with the proportion of B cells in our GIST scRNA-seq cohort (**Figure 2H**). Meanwhile, a significantly higher density of TLS was found in post-imatinib GIST samples compared with pre-imatinib GIST biopsies and GISTs that had undergone only surgery, as determined by mIHC staining (**Figure S2I-J**).

These findings confirmed that GIST patients may benefit from imatinib-based targeted therapy due to remodeling of the TIME, which may be derived from tumor-infiltrating B cells.

### Germinal center responses and antibody class switching to IgG in the GIST TIME

**Figure 3:**
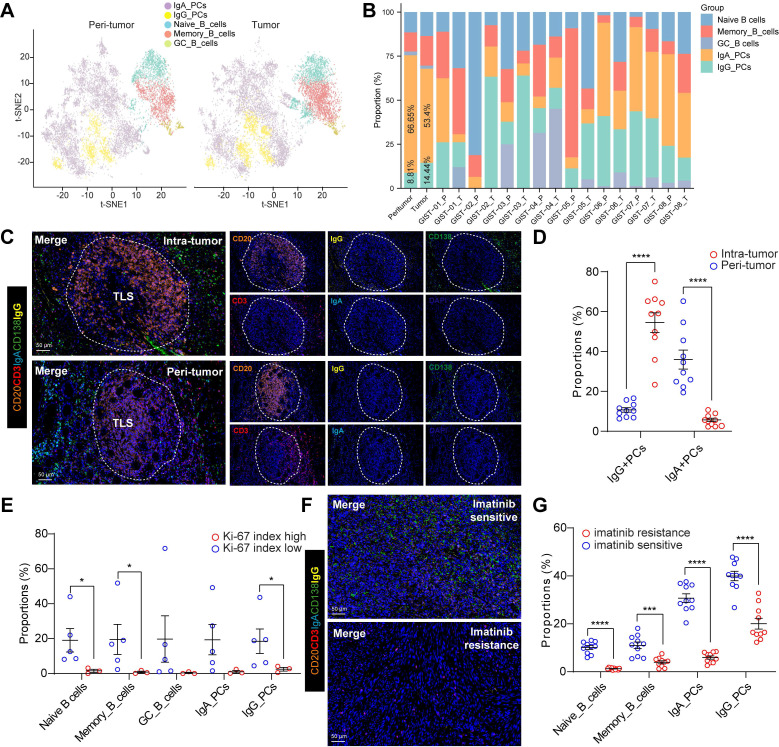
** Germinal center responses and antibody class switching to IgG in the GIST TIME. (A)** t-SNE plot of all B cell subsets between peri-tumor (n = 8) and tumor (n = 8) tissues. **(B)** Proportions of the B cell subsets in GIST peri-tumor (n = 8) and tumor (n = 8) tissues in individual samples. **(C)** Representative mIHC staining of CD3^+^ T cells, CD20^+^ B cells, IgG^+^CD138^+^ PCs, IgA^+^CD138^+^ PCs between peri-tumor and tumor tissues in GIST. Scale bars, 50 μm. **(D)** Dot plots showing the comparison of IgG^+^CD138^+^ PCs, IgA^+^CD138^+^ PCs between peri-tumor (n = 10) and tumor (n = 10) tissues in GIST (Mann-Whitney test). **(E)** Dot plots showing the comparison of B cell subsets between low (n = 5) and high Ki-67 index (n = 3) in GIST (Mann-Whitney test). **(F)** Representative mIHC staining of CD20^+^ B cells, IgG^+^CD138^+^ PCs, IgA^+^CD138^+^ PCs between Imatinib resistance and sensitive GIST. Scale bars, 50 μm. **(G)** Dot plots showing the comparison of B cell subsets between Imatinib resistance (n = 10) and sensitive (n = 10) GIST (Mann-Whitney test). All data were displayed as mean ± SEM. * *P* < 0.05; *** *P* < 0.001; **** *P* < 0.0001.

The presence of intra-tumoral TLSs indicated imatinib sensitivity and a favorable prognosis in GIST patients, prompting us to investigate the potential mechanisms involved. However, the role of TLS in PCs maturation and tumour-specific antibody isotypes in GIST remains unclear. Notably, we detected an increased IgG/IgA PCs ratio in the intra-tumoral tissues from overall (**Figure 3A**) or individual patients (**Figure 3B**) compared with that in peri-tumor tissues. Next, the mIHC staining was performed in GIST specimens to visualize this process. Interestingly, we found that IgG^+^CD138^+^ PCs were present in higher numbers at the periphery of TLS in both intra-tumor and peri-tumor tissues (**Figure 3C**), suggesting that TLS may play a role in PCs maturation and IgG generation. Furthermore, consistent with the scRNA-seq data, a significantly higher proportion of IgG^+^CD138^+^ PCs were generated around TLS in intra-tumor tissues than in peri-tumor tissues (**Figure 3D**). However, compared with the abundant infiltration of IgG^+^CD138^+^ PCs, few IgA^+^CD138^+^ PCs were observed within intra-tumor TLS areas, but more within the peri-tumor TLS areas (**Figure 3C-D**). These findings proved that, unlike IgA^+^CD138^+^ PCs, IgG^+^CD138^+^ PCs were mainly concentrated outside TLS and spread into the tumor stroma of GIST TIME (**Figure S3A**).

We also investigated the correlation between the Ki-67 index and B-cell subsets in GIST patients. Notably, we found that patients with a low Ki-67 index (≤5%) had significantly higher levels of Naive B cells, Memory B cells and IgG PCs than those with a high Ki-67 index (>5%) (**Figure 3E**). Notably, preoperative imatinib resistance GIST presented the lowest proportion of IgG_PCs (0.55%) and GC_B cells (0%), while preoperative imatinib sensitive GIST indicated the highest proportion of IgG_PCs (43.62%) (**Figure 3B, Table S2**). In line with the scRNA-seq data, we further validated the spatial distribution features of the two PCs, with significant more IgG^+^CD138^+^ PCs, but not IgA^+^CD138^+^ PCs in the imatinib sensitive GIST, compared with those in the imatinib resistance GIST tumor tissues (**Figure 3F-G**). Meanwhile, we found that the TLS number was significantly and positively correlated with the proportion of GC_B cells in our GIST scRNA-seq cohort (**Figure S3B**). We speculated that GC_B cells within intra-tumor TLS are more likely to undergo an *in situ* antibody class switch to the IgG antibody isotype.

Together, these results suggest that imatinib-treated GISTs have a unique immune ecosystem. TLS-dominated GC_B cell differentiation and IgG^+^ PC involvement in the humoral immune response may be critical to imatinib-targeted therapy.

### B cell-related cellular interactions within GIST TIME

**Figure 4:**
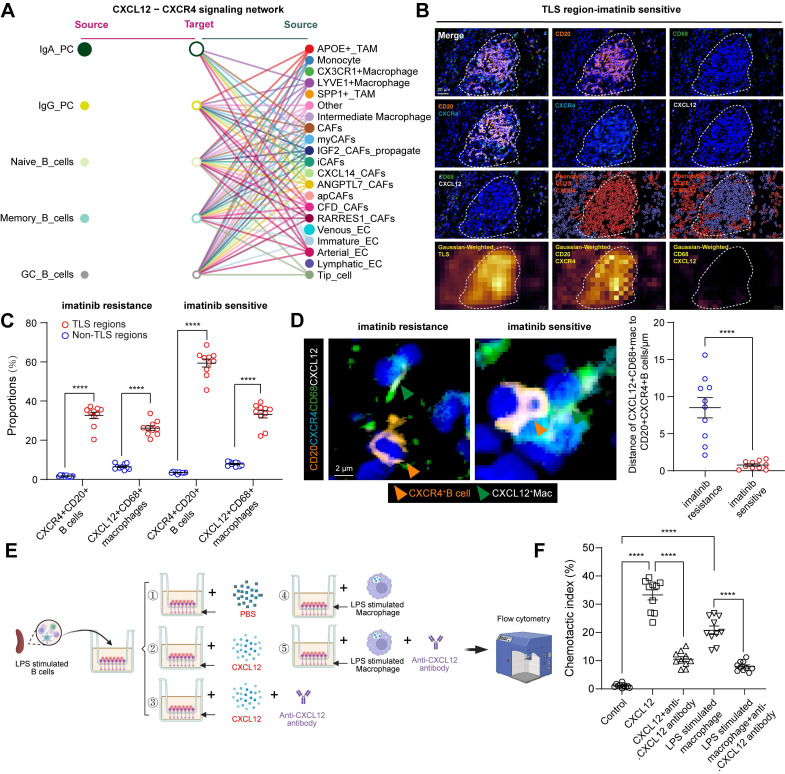
** B cell-related cellular interactions within GIST TIME. (A)** Hierarchical plot shows the inferred intercellular communication network of B cell subsets to other compositions for the CXCL12-CXCR4 signaling networks. **(B)** Representative mIHC staining of CXCR4^+^CD20^+^ B cells and CXCL12^+^CD68^+^ macrophages in TLS region of Imatinib sensitive GIST. HALO phenotype and matched Gaussian-weighted densitogram analysis (Bottom panel) visualizing the co-localization of CXCR4^+^CD20^+^ B cells and CXCL12^+^CD68^+^ macrophages within TLS regions. Scale bars, 20 μm. **(C)** Dot plots showing the comparison of CXCR4^+^CD20^+^ B cells and CXCL12^+^CD68^+^ macrophages between TLS and Non-TLS regions in Imatinib resistance (n = 10) and sensitive (n = 10) GIST (Mann-Whitney test). **(D)** Representative mIHC staining and dot plots showing the distance between CXCR4^+^CD20^+^ B cells (Orange arrow) and CXCL12^+^CD68^+^ macrophages (Green arrow) in Imatinib resistance (n = 10) and sensitive (n = 10) GIST. Scale bars, 2 μm (Mann-Whitney test). **(E)** Flow chart showing the *in vitro* cell chemotaxis experiment by transwell analysis. Migration of LPS stimulated splenic CXCR4^+^ B cells in response to CXCL12-producing macrophages with or without the CXCL12 neutralizing antibody. The chemotactic index was calculated as the ratio of the cell numbers of migrated B cells to that of the total macrophages.** (F)** Dot plots showing the comparison of chemotactic index between different treatments (each group n = 10) (Mann-Whitney test). All data were displayed as mean ± SEM. **** *P* < 0.0001.

The spatial differences between B-cell subsets may be mediated by chemokines and cytokines. We therefore explored the regulation of key chemokine receptors and ligands involved in B-cell trafficking, including CX3CL1-CX3CR1 and CXCL12-CXCR4. Notably, the only signaling network marked as a key trigger for cellular cross-talk between B cells and macrophages using CellChat (**Figure 4A**) was the CXCL12-CXCR4 network, except for CX3CL1-CX3R1 (**Figure S4A**). CXCL12 interacts with its cognate receptor, CXCR4. It plays a critical role in recruiting B cells and forming TLSs 22. We then performed mIHC staining of CD20 and CXCR4 to validate that almost all CD20^+^ B cells expressed the surface marker CXCR4 and that CXCL12 was expressed on CD68^+^macrophages (**Figure 4B**). This was consistent with cell-to-cell interactions and the expression of CXCR4 and CXCR12 in the scRNA-seq data (**Figure S4B-C**). Moreover, a significantly higher number of CXCR4^+^CD20^+^ B cells and CXCL12^+^CD68^+^ macrophages were observed in TLS regions than in non-TLS regions, and in imatinib sensitive GIST than in imatinib resistance GIST (**Figure 4C**).

To confirm their spatial correlations, mIHC staining and Gaussian-weighted densitogram analysis further indicated that, in proximity to TLS regions, CXCL12^+^CD68^+^ macrophages and CD20^+^CXCR4^+^ B cells were co-localized, but were limited in non-TLS regions (**Figure S4D-E**). Analysis of intercellular distances revealed that most CD20^+^CXCR4^+^ B cells were distributed around CXCL12^+^CD68^+^ macrophages, with an average distance of 0.77 μm in imatinib sensitive GIST and 8.51 μm in imatinib resistance GIST. (**Figure 4D**). Meanwhile, a significant positive correlation was found between CXCR4^+^CD20^+^ B cells and CXCL12^+^CD68^+^ macrophages (**Figure S4C**). Furthermore, an *in vitro* chemotaxis assay was performed to validate their spatial distribution features. Consistently, B cells were activated and expressed a significantly higher level of Cxcr4 (GSE35998) following LPS stimulation from 24 to 72 h (**Figure S4D**), while 24 h of LPS stimulation also significantly increased the expression of CXCL12 in macrophages (**Figure S4E**). *In vitro* transwell analysis revealed that CXCL12 and CXCL12-producing CD68^+^ macrophages significantly promote the chemotaxis of CD20^+^CXCR4^+^ B cells. However, this important effect was blocked by anti-CXCL12 neutralizing antibodies (**Figure 4E-F**).

Taken together, these findings suggest that CD68^+^ macrophages have a chemotactic effect on CD20^+^ B cells. This accelerates the formation of TLS via the CXCL12/CXCR4 axis and affects the efficacy of imatinib in GIST.

### B cells and TLS as two immune features of GIC-A tumors and have their clinical implications

**Figure 5:**
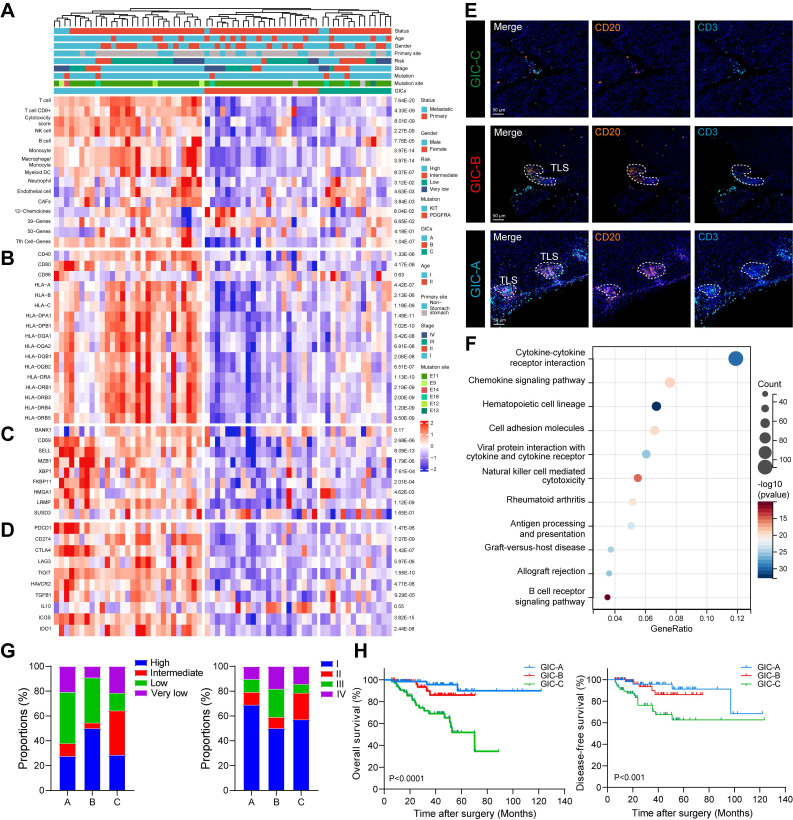
** B cells and TLS as two immune features of GIC-A tumors and have their clinical implications. (A)** Unsupervised consensus clustering analysis of the GIST RNA-seq data (GSE136755) using single sample gene set enrichment analysis (ssGSEA) scores to identify three different GIST immune classes (GICs). Clinical information of each patient is shown on top of the plot. Eight immune and two stromal compositions were identified by GICs. **(B)** Expression of genes related to antigen presentation. **(C)** Expression of genes related to B cell gene markers. **(D)** Expression of genes related to immune checkpoints. **(E)** Representative mIHC staining of three GICs from the human GIST samples. Scale bars, 50 μm. **(F)** Bubble diagram depicting the signaling pathways enriched by KEGG analysis according to upregulated DEGs between GIC-A ad GIC-B tumors. **(G)** Bar chart showing the proportion comparison of tumor risk and stage among three GICs. **(H)** Kaplan-Meier estimates of OS (left panel) and DFS (right panel) in patients by GICs (n = 197).

Next, we investigated the clinical relevance of B cell subsets in GIST. Using eight immune and two stromal cell signature gene sets, we identified three GICs in a cohort of 65 GIST cases (GSE136755) 23. Notably, GIC-A, 'immune infiltrated and activated', showed the greatest abundance of immune cells and moderate stroma cells. GIC-B, 'immune moderate and stroma high', had lower immune and higher stroma profiles than GIC-A. However, GIC-C, 'immune and stroma desert', exhibited the lowest expression levels of immune cells and the lowest abundance of stromal cells (**Figures 5A**). Similar results were confirmed using other immune infiltration analysis tools, including ABIS, CONSENSUS, TIMER, XCELL and ESTIMATE (**Figure S5A**). Consistently, genes related to antigen presentation (CD40, CD80, MHC-I and MHC-II) were significantly and highly elevated in the GIC-A (**Figure 5B**). Furthermore, B cells appeared to be more differentiated into PCs in the GIC-A (**Figure 5C**). Immune checkpoint-related genes (*PDCD1, CD274, CTLA4, ICOS* and *IDO1*) and immunosuppressive-related genes (*TIGIT, HAVCR2* and *LAG3*) were significantly higher in the GIC-A than in the other two groups (**Figure 5D**).

Notably, we found that most B cell subsets were positively and significantly associated with TLS signature (**Figure S5B-C**), which was significantly more elevated in the GIC-A group than in the other two groups (**Figure 5A**). Meanwhile, mIHC staining of CD3^+^ T cells and CD20^+^ B cells in GIST patients further confirmed the presence of three GICs. Furthermore, CD20^+^ B cells within TLS were identified as a distinguishing feature of GIC-A tumors (**Figure 5E**). KEGG pathway analysis consistently showed that GIC-A tumors were characterized by pathways related to the immune response, including the chemokine signaling pathway, cell adhesion molecules (CAMs), cytokine-cytokine receptor interaction and antigen processing and presentation, compared with GIC-B and -C tumors (**Figure 5F, S5D**), highlighting enhanced adaptive immunity in GIC-A tumors. Similar results were also observed when comparing the high and low TLS signature groups (**Figure S5E**).

By integrating the three GICs with clinical features, no clear associations with age, gender or primary tumour site were found (**Figure S5F-H**). However, GIC-A tumors were predicted to be diagnosed at an early stage and within the low-risk category (**Figure 5G**). Moreover, survival analyses indicated favorable survival for the immune-enriched group (GIC-A), which was consistent with STS. However, the immune desert group (GIC-C) was associated with an unfavorable prognosis for both overall survival (OS) and disease-free survival (DFS) in our GIST tissue cohort (**Figure 5H**).

Collectively, the GIC-A group was defined as 'immune-inflamed' or 'hot tumors', indicating antigen activation, the activation of adaptive immunity, and a favourable prognosis.

### GIC-A tumors predicted target and immune therapy

The presence of a tumour kit mutation is known to be associated with a better response to imatinib treatment 24. Thus, we investigated the relationship between *KIT/PDGFRA* and GICs. A total of 65 GIST cases (29, 22 and 14 patients in GIC-A, GIC-B and GIC-C, respectively) with complete mutation profiling were enrolled (**Figure 5A**). The mutational landscape revealed a higher frequency of *KIT* Exon 11 mutations in GIC-A and GIC-B (88.89% and 95.23%, respectively) than in GIC-C (76.9%). This may explain why they responded more favorably to imatinib treatment. Consistently, the mIHC staining further validated that the frequency of *KIT* Exon 11 mutations was highest in the GIC-A group (69.3%), followed by the GIC-B group (59.4%) and the GIC-C group (62.1%).

**Figure 6:**
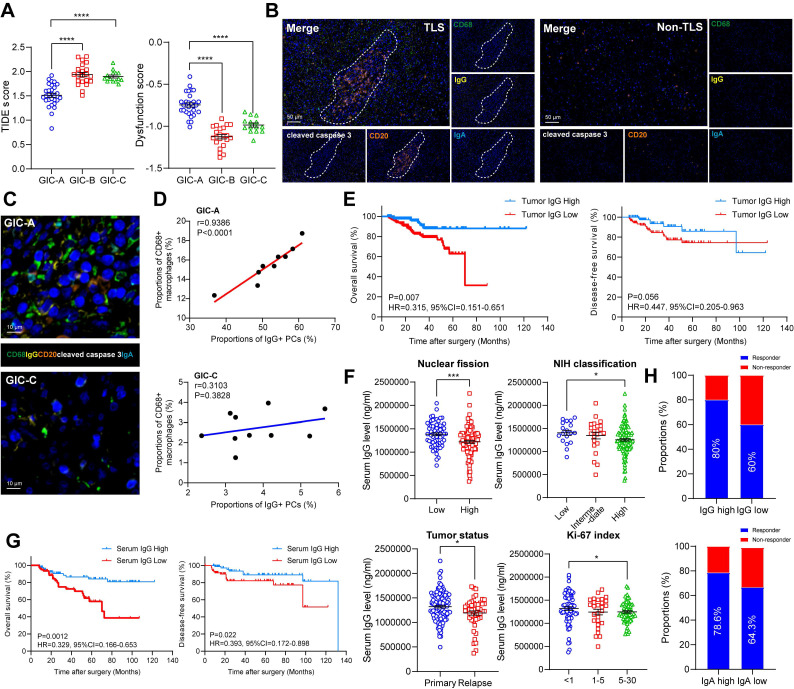
** GIC-A tumors predicted target and immune therapy. (A)** Dot plots showing the comparison of TIDE and dysfunction scores among three GIC groups (unpaired t test). **(B)** Representative mIHC staining showing CD68^+^ macrophages and IgG-stained apoptotic cleaved caspase 3^+^ tumor cells between TLS and Non-TLS regions. Scale bars, 50 μm. **(C)** Representative mIHC staining showing CD68^+^ macrophages and IgG-stained apoptotic cleaved caspase 3^+^ tumor cells between GIC-A and -C groups. Scale bars, 10 μm. **(D)** Spearman correlation between CD68^+^ macrophages and IgG^+^ PCs in GIC-A (n = 10) and GIC-C (n = 10) groups. **(E)** Kaplan-Meier estimates of OS (left panel) and DFS (right panel) in patients with high and low tumor IgG staining (n = 197). **(F)** Dot plots showing the comparison of serum IgG levels between low and high nuclear fission, NIH classification, tumor status, and Ki-67 index (unpaired t test). **(G)** Kaplan-Meier estimates of OS (left panel) and DFS (right panel) in patients with high and low serum IgG levels (n = 169). **(H)** Bar chart showing the proportion comparison of Responder and Non-Responder for preoperative Imatinib treatment based on high and low serum IgG and IgA levels. All data were displayed as mean ± SEM. * *P* < 0.05; *** *P* < 0.001; **** *P* < 0.0001.

Since tumors with immune infiltration and activation showed higher sensitivities to immune checkpoint inhibitor (ICI) treatment 25,26, we further evaluated the predicted response of GIC-A patients to ICB treatment in GSE136755. Notably, GIC-A tumors had significantly lower TIDE scores and exclusion scores, but significantly higher IFNG expression, and dysfunction scores (**Figure 6A, S6A**), indicating that GIC-A tumors are more sensitive to ICI treatment than GIC-B and -C groups. However, no significant differences were observed in the MSI expression signature scores of GICs (**Figure S6B**), which further highlights the fundamental differences between GIST and epithelial tumors, namely the absence of obvious genomic features such as microsatellite instability 27.

The accumulation of IgG resulted in a pronounced inflammatory phenotype characterized by elevated levels of proinflammatory cytokines, which are considered to be functional markers of the TLS niche 28. Meanwhile, the presence of TLS and Ig-producing PCs was associated with a greater number of IgG antibodies binding to apoptotic tumour cells and a better response to immunotherapy 29. Having scrutinized the role of B cells and TLS formation in the GIC-A tumors, we further endeavoured to investigate the presence and functionality of immunoglobulins in the clinical setting through our GIST tissue cohort. Consistent with the TLS findings in **Figure 3C**, the mIHC staining data showed that IgG^+^ and CD68^+^ macrophages were more prevalent in the presence of TLS but scarce in its absence (**Figure 6B, S6C-D**). The proximity assessment further emphasized the positive relationship between CD68^+^ macrophages and IgG-stained apoptotic cleaved caspase 3+ tumor cells in GIC-A tumors, but not in GIC-C tumors (**Figure 6C-D**). This further highlights that IgG, which is secreted by TLS-PCs, may exert an anti-tumour immune function through the ADCP effect 29,30, particularly in GIC-A tumors.

To evaluate the activity of enhanced adaptive immunity therapeutics in ADCP, an *in vitro* experiment was conducted using a co-culture of macrophages and GIST tumor cells, some of which were stimulated with imatinib and some of which were not (**Figure S6E**). Notably, flow cytometry analysis revealed that macrophages (labeled by Calcein AM/FITC) captured the highest proportion of tumour cells (labeled by CypHer5E/APC), a process that was significantly reduced by FcR receptor blockade (**Figure S6F**).

Further corresponding survival analysis confirmed that an enhanced adaptive immune response, characterized by an accumulation of IgG, significantly prolonged OS in GIST tumors, but did not affect DFS (**Figure 6E**). Moreover, tumor with low nuclear fission (<5 HPF), a lower NIH classification and a low Ki-67 index had significantly higher serum IgG levels (**Figure 6F**), but not IgA. However, both serum IgG and IgA levels were significantly higher in primary GIST than those in recurrent GIST (**Figure S6H**). Consistently, a high level of serum IgG, but not IgA, was associated with favorable OS and DFS compared with the low groups (**Figure 6G, S6I**). To further clarify the independent risk factors, we conducted univariate and multivariate Cox regression analyses. Notably, the univariate analysis revealed that tumor number, tumor texture, mitotic index, NIH grading, WHO prognosis group, Ki-67 index, tumor status and serum IgG level were significantly associated with OS (all *P* < 0.05). However, none of these values were significant in the multivariate analysis, highlighting the interference between clinical and pathological factors (**Table S9**).

To explore the impact of IgG accumulation on imatinib-treated patients, we analyzed 29 available tumors from imatinib-treated patients. We selected mean serum IgG (50%, 1,229,538.4 ng/ml) and IgA (50%, 31,629.035 ng/ml) levels as the cut-off points. Among imatinib-treated patients, we found a significant association between therapeutic responses and serum IgG and IgA levels above the mean: 80% (12/15) and 78.6% (11/14) of patients, respectively, compared to 60% (9/15) and 64.3% (9/14) with serum IgG levels below the mean (**Figure 6H**).

These results reinforce the idea that cycling and the accumulation of IgG within tumors play a pivotal role in the sensitivity of immunotherapy and imatinib-targeted therapy. They also suggest that interventions that induce IgG expression could improve the prognosis of GISTs.

## Discussion

The role of CD8^+^ T cells and their distinct subsets in GIST has been thoroughly explored 11-13. Current immunotherapies primarily target dysfunctional and exhausted T cells, which play a vital role in the immune system 31,32. However, the contribution of B cells has been studied much less extensively. This study uses a combination of high-resolution single-cell transcriptomics and spatial mIHC staining to analyse the GIST TIME. We demonstrate that the GIST TIME is a heterogeneous ecosystem in terms of both structure and function, and that targeted KIT inhibition arrests oncogenic signaling and orchestrates TLS neogenesis. GC_B cell differentiation within TLSs and the subsequent emergence of IgG^+^ PCs constitute an under-appreciated determinant of imatinib sensitivity. We also reveal a CXCR4/CXCL12-dependent interaction whereby CD68^+^ macrophages attract CD20^+^ B cells to initiate TLS formation. Finally, we reveal that intra-tumoral IgG antibodies are not merely inert bystanders, but actively shape both imatinib and ICI responsiveness. We also show that the deliberate induction of IgG production may represent a rational combinatorial strategy.

Historically, GIST has been considered an immunologically 'cold' sarcoma, characteriszed by a dominance of myeloid suppressor cells and a scarcity of T-cell infiltrates 33,34. Our scRNA-seq atlas of 15,3515 immune cells from 6 treatment-naïve and 2 imatinib-treated tumors now challenges this dogma. By integrating gene-expression data, we identify 13 distinct immune subpopulations. This heterogeneity aligns with recent pan-cancer TIME taxonomies 35, but is remarkable given GIST's low tumor mutational burden 27. The immunogenicity of GISTs may therefore be less dependent on the quantity of neoantigens and more dependent on the context-dependent presentation of *KIT* exon 11 neo-peptides. We propose that TLS act as ectopic lymphoid niches that amplify sub-threshold antigens, generating robust adaptive responses.

TLSs are traditionally associated with prolonged survival in solid tumors 36. In sarcomas, TLS may serve as a favorable prognostic biomarker in STS 37,38, alveolar soft part sarcoma (ASPS) 39 and undifferentiated pleomorphic sarcoma (UPS) 40. Furthermore, the presence of TLS in advanced STS is a potential predictive biomarker that could be used to select patients for Pembrolizumab treatment 41,42. In GIST, concomitant with LIGHT-mediated vascular remodeling, intratumoral high endothelial venules (HEVs) and TLS were formed in the cord blood humanized mouse models, resembling the spontaneous TLS found in GIST patients 43. However, most of these studies have focused primarily on the clinical cohort analysis of TLS and its prognostic value, rather than on the underlying mechanisms of its formation, maturation or function. Our previous data extend this paradigm by demonstrating that imatinib efficacy is proportional to TLS maturity 44. Spatial transcriptomics reveals that TLS+ tumors upregulate IFN-γ and CXCL13 within 5 μm of GC_B-cell follicles 44, recapitulating the “immune-active” signature predictive of imatinib response. Computational depletion of B cells reduced predicted imatinib sensitivity scores by 34%, highlighting a causal rather than a correlative relationship 12. Collectively, TLSs emerge as dynamic reaction centers where imatinib-induced tumor stress antigens are efficiently translated into humoral immunity, which in turn enhances targeted therapy. We therefore posit that TLS-derived PCs serve dual roles: (i) the local secretion of tumor-reactive IgG within the TIME, and (ii) systemic dissemination of antibodies that target circulating tumor cells, thereby limiting metastatic seeding.

Deleting Cxcl12 from Osterix-expressing stromal cells (including CXCL12-abundant reticular cells and osteoblasts) results in the constant movement of haematopoietic progenitor cells (HPCs) and a loss of B-lymphoid progenitors 45. Our mIHC data revealed a subset of CD68^+^ macrophages that co-express CXCL12 and are located in the TLS mantle zone. *In vitro* transwell assays confirmed that recombinant CXCL12 induced the migration of CD20^+^ B cells, an effect that was abrogated by a CXCL12 antibody.

Given that upregulation of IgG in tumor areas correlates with ICI immune-therapy 29, and that our study indicated that GIC-A tumors presented significantly lower TIDE scores and exclusion scores, but significantly higher IFNG expression, and dysfunction scores. We then hypothesized that TLS-rich GIST may be primed for ICI treatment. Mechanistically, IgG immune complexes bind FcγR on dendritic cells, thereby enhancing cross-presentation and T-cell reinvigoration 46,47. Future trials should stratify patients by IgG density and TLS maturity in order to refine ICI-GIST combination strategies.

Our findings align with the “immunogenic cell death” paradigm, in which targeted therapies remodel the TIME 48. However, in contrast to breast cancer where CD40 agonists enhance TLS 49, GIST TLS are spontaneously amplified by imatinib. Furthermore, whereas previous studies have emphasized T-cell exhaustion 50, we highlight B-cell-mediated humoral immunity as an additional axis.

There are several caveats that merit attention. Firstly, we found that only a limited number of paired pre- and post-treatment GIST samples could be enrolled for scRNA-seq in this study. The small number of imatinib treatment samples also restricted the quality of immune profiles. Secondly, our cohorts were enriched for gastric GIST, which may limit generalizability to small intestinal tumors. Thirdly, further murine modelling is required to establish causality, as even species differences in TLS biology necessitate validation in humanized mice or patient-derived explants. Meanwhile, the *in vitro* transwell assays of CD68^+^ macrophages induced chemotactic effects on CD20^+^ B cells, thus promoting TLS formation through the CXCL12/CXCR4 axis. However, this required further validation in *in vivo* experiments. Due to limitations in using animal models to study GIST, and the inability of PDX models to accurately reflect the TIME and clinical state of GIST disease, we cannot currently validate the *in vivo* chemotactic effect of CD68^+^ macrophages on CD20^+^ B cells, which promotes TLS formation via the CXCL12/CXCR4 axis. Subsequent work will employ PDX models combined with humanized immune systems for validation and further exploration. Finally, the collective calibration of CXCL12 output by macrophage metabolic states, epigenetic landscapes, splice-isoform selection, pulsatile secretion dynamics and TLS architecture consolidation by reciprocal B-cell chemokine feedback and fibroblast cross-talk all require further exploration.

In summary, our study sheds light on the landscape of infiltrating B cells and their associated clinical outcomes in GIST. A better understanding of B cell characteristics will improve our knowledge of their role in TIME and facilitate the development of novel immunological strategies that targeting B cells.

## Supplementary Material

Supplementary figures and tables.
