# Supplementary material for: Enhanced formation of tertiary lymphoid structures shapes the anti-tumor microenvironment in gastrointestinal stromal tumors after imatinib targeted therapy
Source: Theranostics. 2026 Jan 1;16(6):2829–44. doi: 10.7150/thno.123923 (PMC12775820; doi:10.7150/thno.123923)
Supplement: Supplementary file 1 — Supplementary figures and tables. [file thnov16p2829s1.pdf]

Supplementary materials

Enhanced formation of tertiary lymphoid structures shapes the anti-tumor microenvironment in gastrointestinal stromal tumors after Imatinib targeted therapy

Supplementary Figure and Figure legends

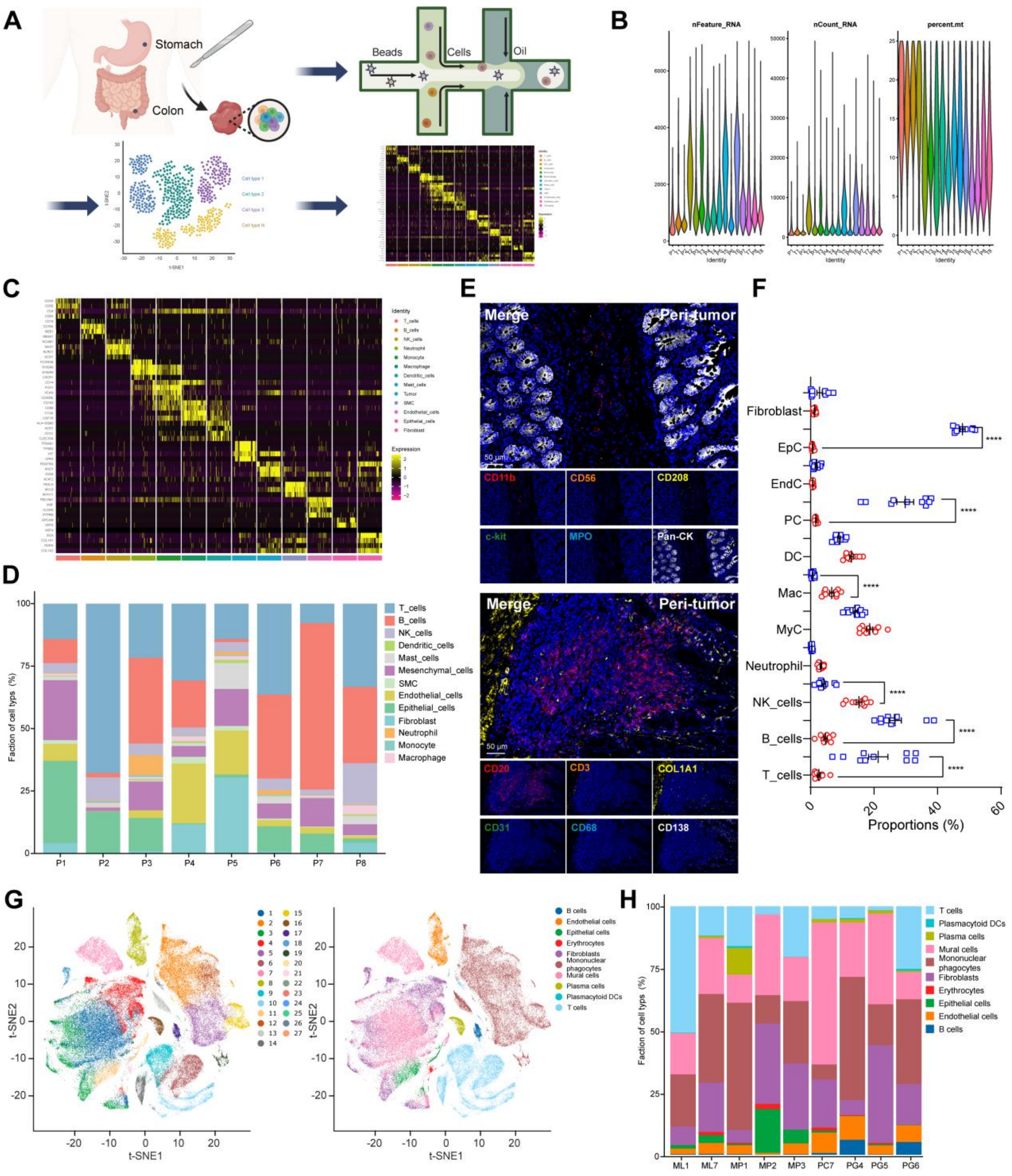

Figure S1. High-resolution landscape of the tumoral ecosystem in GIST

by single cell profiling. (A). Study overview. Resected tumor tissues were digested to single-cell suspensions, and subjected to single-cell assays shown. **(B).** Single-cell data quality control. **(C).** Expression of cell-type marker genes across immune scRNA-seq clusters. Heatmap shows the average expression per cell. Clusters are shown using even sampling of cells from eight patients. **(D).** Proportions of the immune scRNA-seq clusters in GIST peri-tumor tissue in individual samples. **(E).** Representative mIHC staining of $CD3^+$ T cells, $CD20^+$ B cells, $CD31^+$ endothelial cells, $CD68^+$ macrophages, $CD138^+$ PCs, $COL1A1^+$ fibroblasts, $CD11b^+$ myeloid cells, $CD56^+$ NK cells, $CD208^+$ dendritic cells, c-kit$^+$ mast cells, MPO^+ neutrophils, and Pan-CK$^+$ epithelial cells in GIST peri-tumor tissues. Scale bars, 50 μm. **(F).** Dot plots showing the proportions of immune scRNA-seq clusters between peri-tumor (n = 8) and tumor (n = 8) tissues (Mann-Whitney test). **(G).** t-SNE plot of all immune scRNA-seq clusters in the public scRNA-seq dataset (n = 7, GSE254762), with each colour representing one cluster. **(H).** Proportions of the immune scRNA-seq clusters in GIST tumor tissue in individual samples (n = 7) (ML: GIST liver metastasis; MP: GIST peritoneal metastasis; PG: primary GIST; GSE254762). All data were displayed as mean ± SEM. **** $P < 0.0001$.

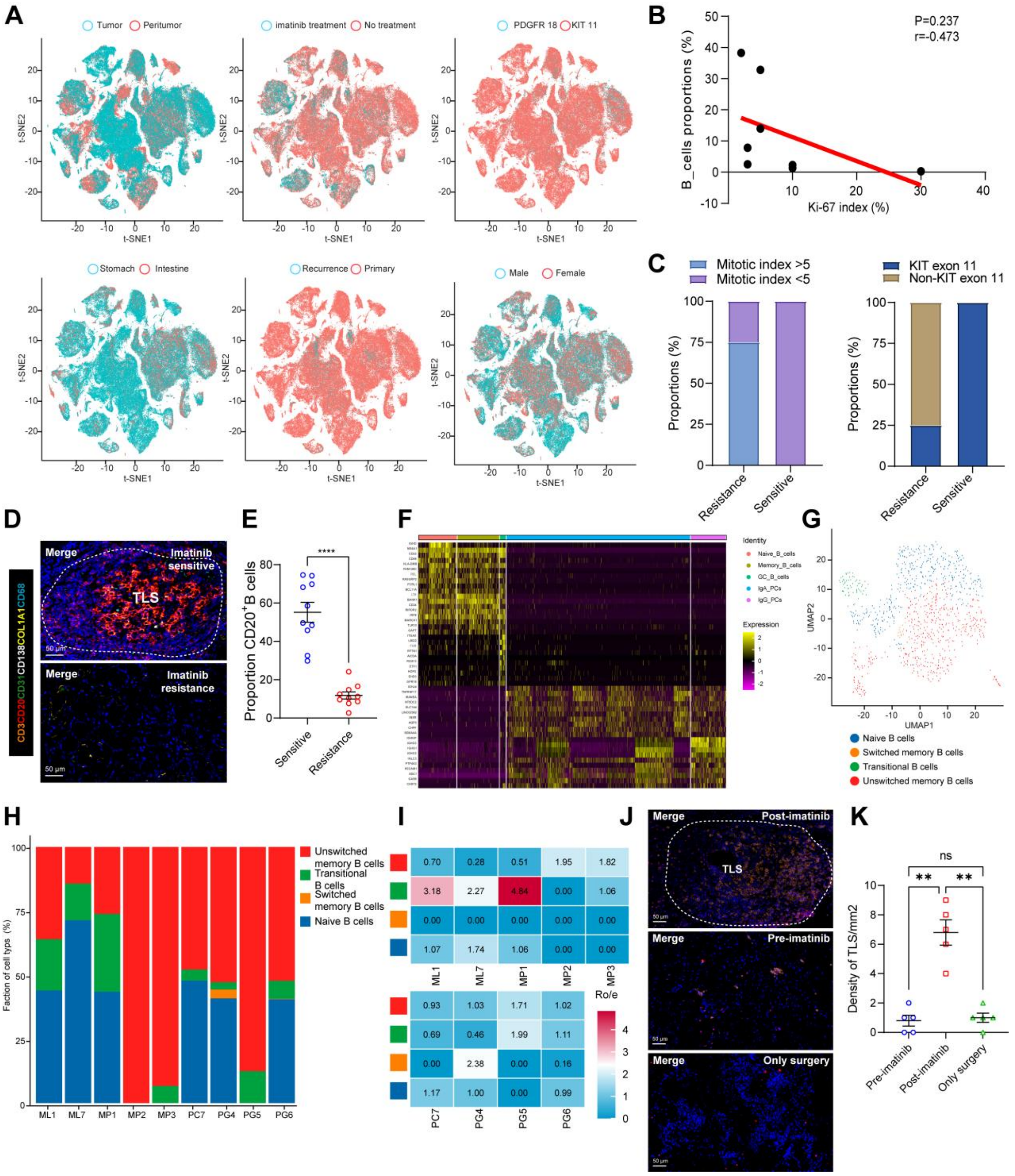

Figure S2. Spatial infiltration features of B cell subsets in GIST with Imatinib targeted therapy. (A). t-SNE plot showing the comparison of immune scRNA-seq clusters between different clinical features. **(B).** Spearman correlation between B cell proportions and Ki-67 index in the scRNA-seq cohort (n = 8). **(C).** Bar chart showing the proportion comparison of mitotic index between Imatinib resistance and sensitive GIST in the public scRNA-seq cohort (n = 7, GSE254762). **(D).** Representative mIHC staining of CD3⁺ T cells, CD20⁺ B cells, CD31⁺ endothelial cells, CD68⁺ macrophages, CD138⁺ PCs, and COL1A1⁺ fibroblasts in peri-tumor tissues between Imatinib resistance

and sensitive GIST. Scale bars, 50 µm. **(E).** Dot plots showing the comparison of CD20$^+$ B cells in peri-tumor tissues between Imatinib resistance (n = 10) and sensitive (n = 10) GIST (Mann-Whitney test). **(F).** Expression of cell-type marker genes across B cell clusters. Heatmap shows the average expression per cell. Clusters are shown using even sampling of cells from eight patients. **(G).** UMAP plot of four B cell subsets in the public scRNA-seq cohort (n = 7, GSE254762). **(H).** Proportions of the B cell subsets in GIST in individual samples (n = 7, GSE254762). **(I).** Tissue group preference of each B cell subsets measured by the ratio of observed to randomly expected cell numbers (RO/E) calculated by the STARTRAC-dist algorithm. **(J).** Representative mIHC staining of CD3$^+$ T cells, CD20$^+$ B cells for TLS identification between pre-Imatinib, post-Imatinib and only surgery tumor tissues. **(K).** The comparison of TLS density between pre-Imatinib (n = 5), post-Imatinib (n = 5) and only surgery (n = 5) tumor tissues (Mann-Whitney test). All data were displayed as mean ± SEM. ns $P > 0.05$; **** $P < 0.0001$.

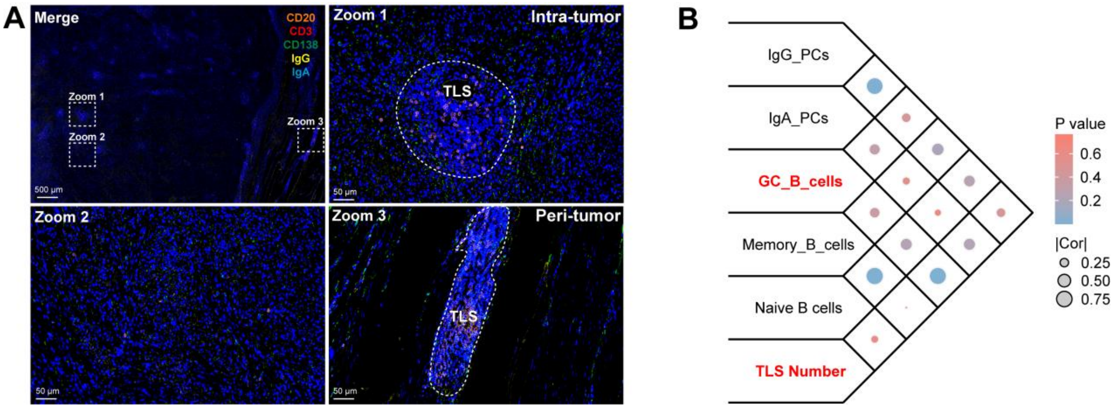

Figure S3. Germinal center responses and antibody class switching to IgG in the GIST TIME. (A). Representative mIHC staining showing the distribution features of CD3$^+$ T cells, CD20$^+$ B cells, IgG$^+$CD138$^+$ PCs, IgA$^+$CD138$^+$ PCs between TLS and Non-TLS regions in GIST. Scale bars, 500 µm, Zoom in 50 µm. **(B).** Bubble diagram depicting the spearman correlation between TLS number and B cell clusters in the GIST scRNA-seq cohort.

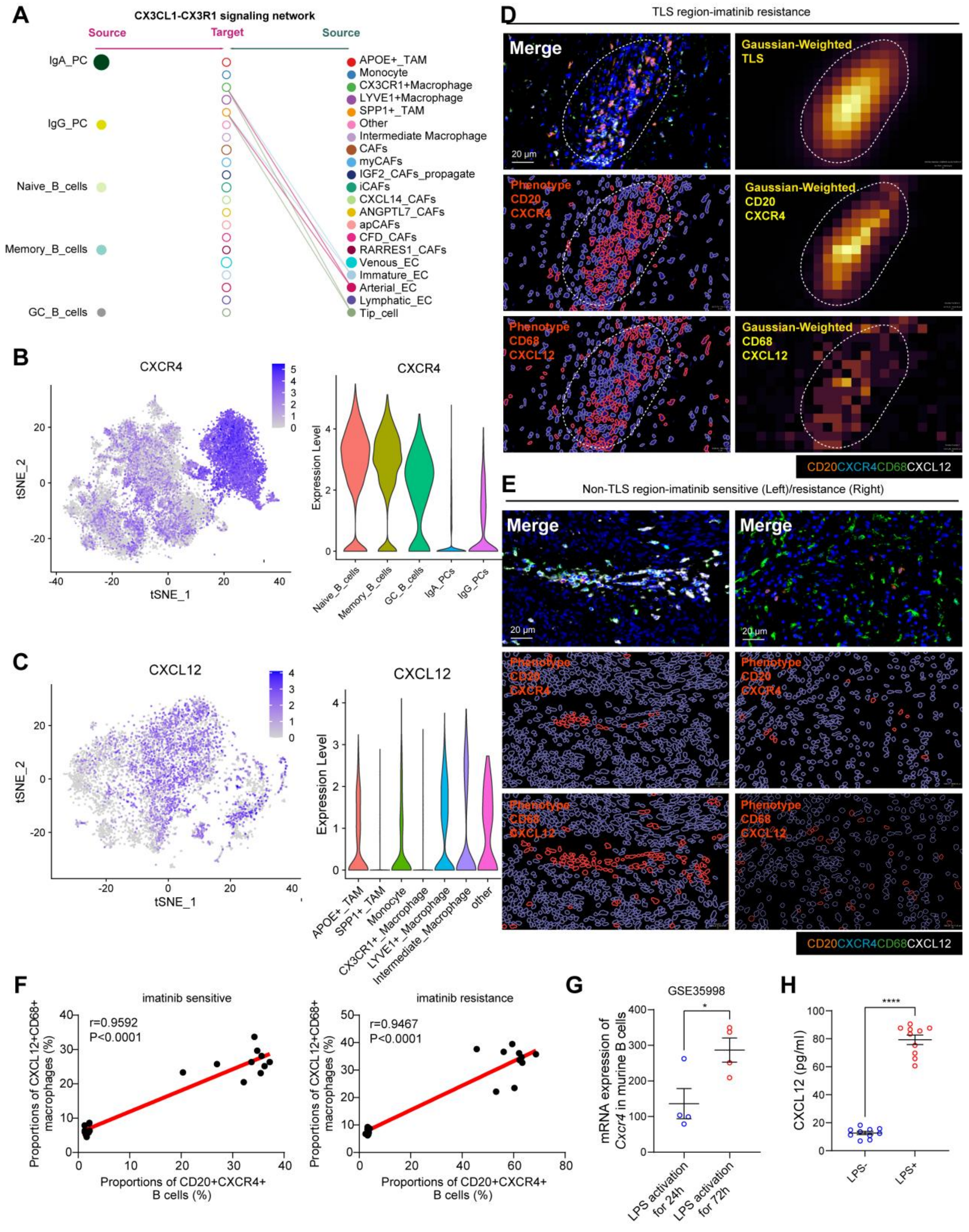

Figure S4. B cell-related cellular interactions within GIST TIME. (A). Hierarchical plot shows the inferred intercellular communication network of B cell subsets to other compositions for the CX3CL1-CX3R1 signaling networks. **(B).** tSNE map and violin diagram showing the mRNA expression of CXCR4 in all fiver B cell subsets. **(C).** tSNE map and violin diagram showing the mRNA expression of CXCL12 in all macrophage subsets. **(D).** Representative mIHC

staining of $CXCR4^+CD20^+$ B cells and $CXCL12^+CD68^+$ macrophages in TLS region of Imatinib resistance GIST. HALO phenotype and matched Gaussian-weighted densitogram analysis (right panel) visualizing the co-localization of $CXCR4^+CD20^+$ B cells and $CXCL12^+CD68^+$ macrophages within TLS regions. Scale bars, 20 μm. **(E).** Representative mIHC staining of $CXCR4^+CD20^+$ B cells and $CXCL12^+CD68^+$ macrophages in Non-TLS region of Imatinib sensitive (left)/resistance (right) and matched HALO phenotype (bottom panel) visualizing the co-localization of $CXCR4^+CD20^+$ B cells and $CXCL12^+CD68^+$ macrophages. Scale bars, 20 μm. **(F).** Spearman correlation between $CXCR4^+CD20^+$ B cells and $CXCL12^+CD68^+$ macrophages in Imatinib sensitive (left)/resistance (right) GIST. **(G).** Dot plots showing the comparison of mRNA expression of *Cxcr4* on B cells between LPS stimulated for 24h and 72h in GSE35998 (Mann-Whitney test). **(H).** Dot plots showing the comparison of CXCL12 levels of macrophages detected by ELISA between LPS stimulated or not (Mann-Whitney test). All data were displayed as mean ± SEM. * $P < 0.05$; **** $P < 0.0001$.

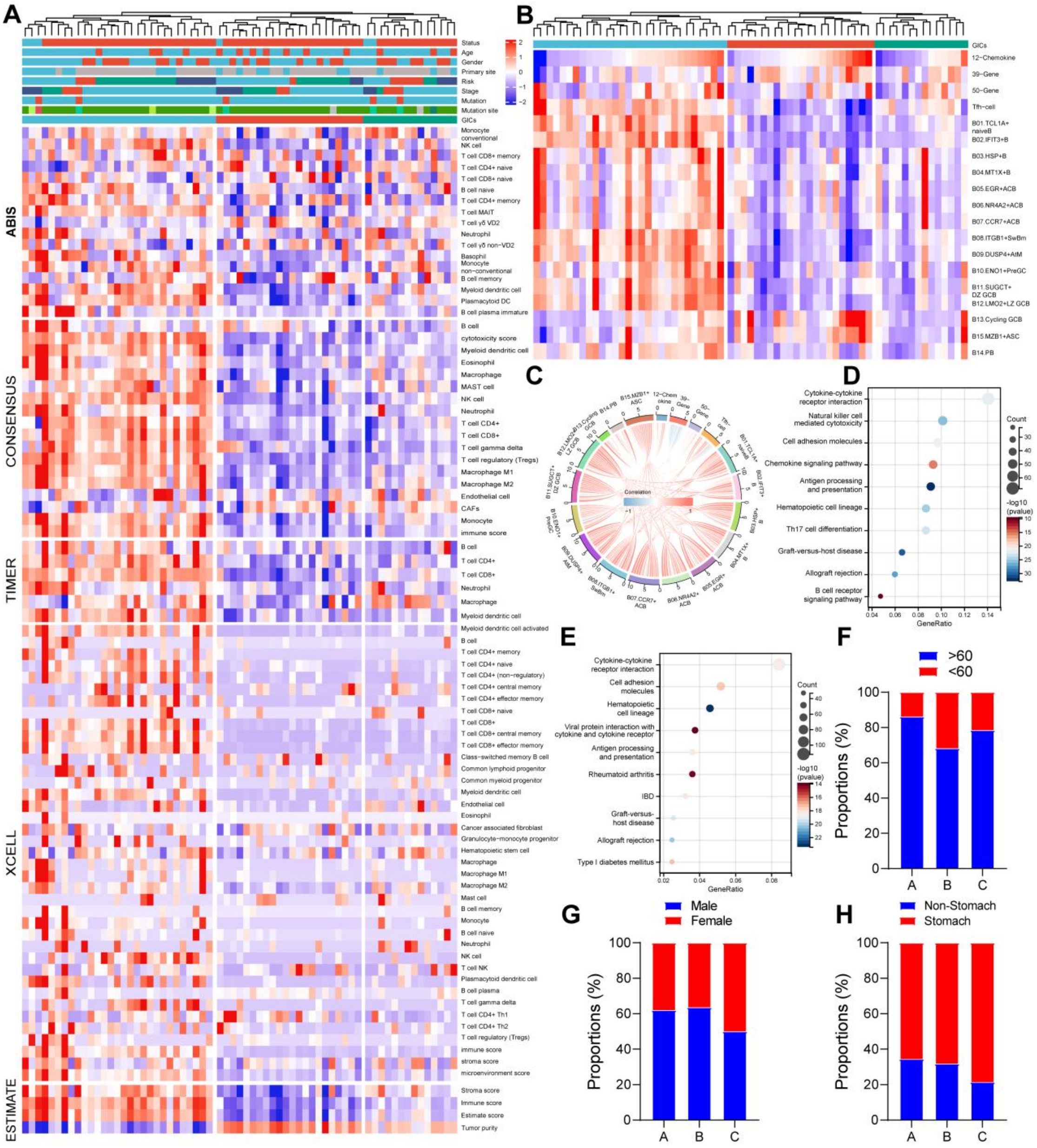

Figure S5. B cells and TLS as two immune features of GIC-A tumors and have their clinical implications. (A). Unsupervised consensus clustering analysis of the GIST RNA-seq data (GSE136755) using single sample gene set enrichment analysis (ssGSEA) scores to identify three different GIST immune classes (GICs). Clinical information of each patient is shown on top of the plot. Eight immune and two stromal compositions were identified by GICs. Expression of gene signature performed by ABIS, CONSENSUS, TIMER, XCELL, and ESTIMATE. **(B).** Expression of gene signature performed by validated TLS signatures and B cell subsets. **(C).** Spearman correlation between validated TLS signatures and B cell subsets signature scores. **(D).**

Bubble diagram depicting the signaling pathways enriched by KEGG analysis according to upregulated DEGs between GIC-A ad GIC-C tumors. **(E).** Bubble diagram depicting the signaling pathways enriched by KEGG analysis according to upregulated DEGs between high and low TLS signature tumors. **(F-H)**. Bar chart showing the proportion comparison of Bar chart showing the proportion comparison of age (**F**), gender (**G**), and tumor site (**H**) among three GICs.

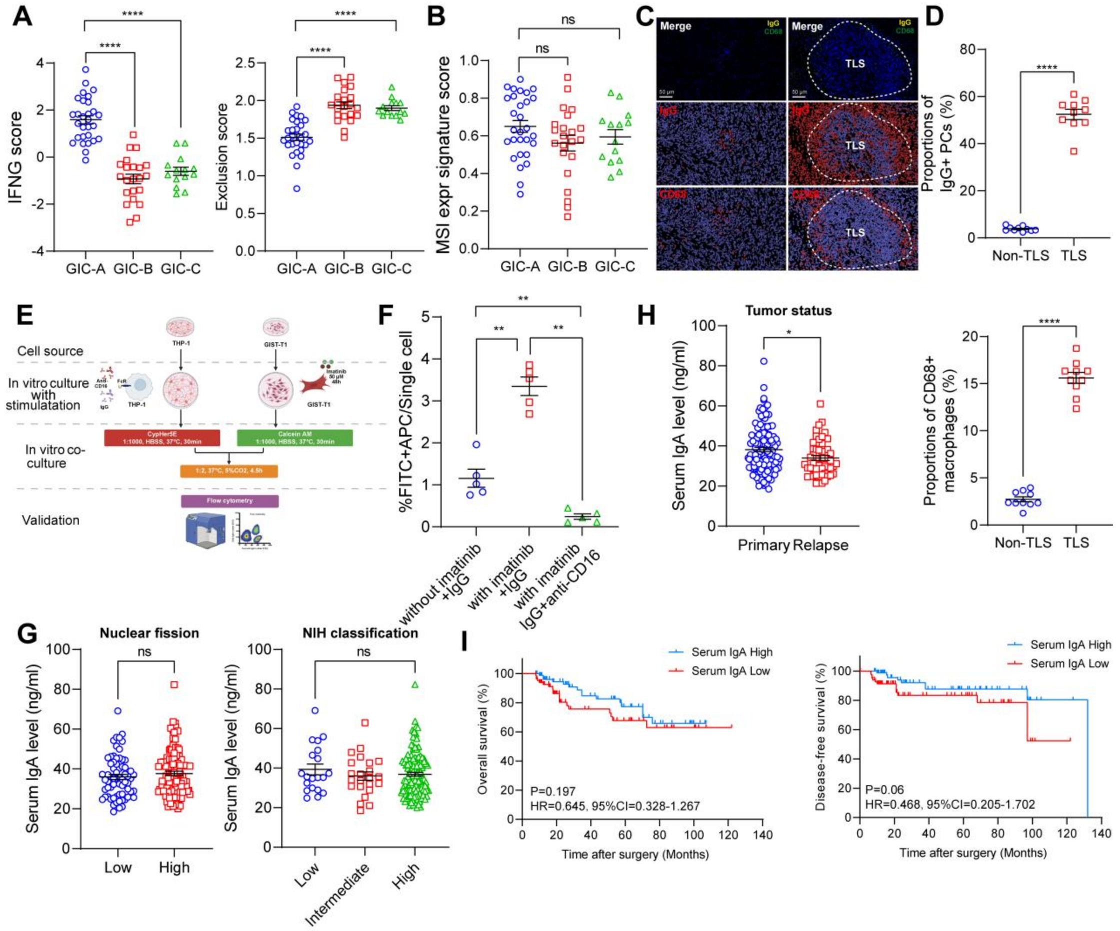

Figure S6. GIC-A tumors predicted target and immune therapy. (A). Dot plots showing the comparison of IFNG and exclusion scores among three GIC groups (unpaired t test). **(B).** Dot plots showing the comparison of MSI expr signature among three GIC groups (unpaired t test). **(C).** Representative mIHC staining showing CD68⁺ macrophages and IgG⁺ PCs between TLS and Non-TLS regions. Scale bars, 50 μm. **(D).** Dot plots showing the comparison of

$CD68^+$ macrophages and IgG^+ PCs between TLS (n=10) and Non-TLS (n=10) regions (Mann-Whitney test). **(E).** Flow chart showing the ADCP experiment by *in vitro* cell co-culture and flow cytometry analysis. **(F).** Dot plots showing the comparison of FITC+APC+ cells between without Imatinib treatment, with Imatinib treatment, and with Imatinib combined with anti-CD16 treatment (Mann-Whitney test). **(G).** Dot plots showing the comparison of serum IgA levels between low and high nuclear fission, NIH classification (unpaired t test). **(H).** Dot plots showing the comparison of serum IgA levels between primary and relapse GISTs (unpaired t test). **(I).** Kaplan-Meier estimates of OS (left panel) and DFS (right panel) in patients with high and low serum IgA levels (n=169). All data were displayed as mean ± SEM. ns $P > 0.05$; ** $P < 0.01$; **** $P < 0.0001$.

Table S1-S9

Table S1. Detailed clinical and pathological information of GIST tissue cohort (n=197).

Characteristics	Tissue cohort (n=197)
**Gender**	
Male	109 (55.3%)
Female	88 (44.7%)
**Age**	
<60	96 (48.8%)
≥60	101 (51.2%)
**Nuclear fission**	
<5	93 (47.2%)
≥5	104 (52.8%)
**Pathologic subtypes, n (%)**	
Epithelioid	12 (6%)
Spindle	165 (83.7%)
Mixed	30 (10.3%)
**NIH grading, n (%)**	
Extremely low risk	4 (2%)
Low risk	37 (18.7%)

Moderate risk	27 (13.7%)
High risk	129 (65.6%)
**Tumor diameter, cm**	
<5	72 (36.5%)
≥5	125 (63.5%)
**Multifocality**	
No	134 (68%)
Yes	63 (32%)
**Combined organ resection, n (%)**	
Simple tumor resection	2 (1%)
Tumor and gastrointestinal tract resection	132 (67%)
Tumor, gastrointestinal tract, and extra organ resection	63 (32%)
**Tumor texture**	
Hard	155 (78.6%)
Soft	42 (21.4%)
**Transfusion**	
No	169 (85.8%)
Yes	28 (14.2%)

**Tumor classification**	
Primary	163 (82.7%)
Recurrence	34 (17.3%)
**Tumor site, n (%)**	
Gastric	112 (56.8%)
Intestine	85 (43.2%)
**Mutation site, n (%)**	
Non kit-11	47 (23.9%)
kit-11	125 (63.4%)
PDGFRA	9 (4.6%)
other	16 (8.1%)
**Ki-67 index (%)**	
<15	144 (73%)
≥15	53 (27%)
**Preoperative WBC (10^9/L), Mean ± SD**	5.76 ± 2.87
**Preoperative RBC (10^9/L), Mean ± SD**	3.96 ± 0.72
**Preoperative Neutrophils (10^9/L), Mean ± SD**	4.1 ± 5.85
**Preoperative Lymphocyte (10^9/L), Mean ± SD**	1.54 ± 1.38

**Preoperative Monocyte (10^9/L), Mean ± SD**	0.44 ± 0.27
**Preoperative PLT (10^9/L), Mean ± SD**	232.1 ± 112.4

Table S2. Detailed clinical and pathological information of eight GIST patients for scRNA-seq analysis.

Number	Sample	Gender	Age	Location	Mutation site	Preoperative Imatinib	Imatinib resistance	Tumor status	Tumor size (cm)	NIH grading	Ki-67 (%)	Imatinib treatment cycle	Intra-tumor TLS Number
1	Tumor/peri-tumor	Male	62	Stomach	PDGFRA 18 exons	No	-	Primary	10	High risk	5		0
2	Tumor/peri-tumor	Male	63	Small intestine	KIT 11 exons	Yes	Yes	Recurrence	8	High risk	30	400mg (po, qd) for five years; 200mg(po, qd) for seven years	0

3	Tumor/peri-tumor	Female	42	Stomach	KIT 11 exons	No	-	Primary	9	High risk	10		0
4	Tumor/peri-tumor	Male	65	Stomach	KIT 11 exons	No	-	Primary	4	Low risk	5		5
5	Tumor/peri-tumor	Male	74	Stomach	KIT 11 exons	No	-	Primary	4	Moderate risk	3		2
6	Tumor/peri-tumor	Female	53	Stomach	KIT 11 exons	No	-	Primary	6	Moderate risk	10		3
7	Tumor/peri-tumor	Male	68	Stomach	KIT 11 exons	Yes	No	Primary	7	Low risk	2	400mg (po, qd) for four months	25
8	Tumor/peri-tumor	Female	70	Small intestine	KIT 11 exons	No	-	Primary	4	High risk	3		0

Table S3. Detailed clinical and pathological information of GIST serum cohort (n=169).

Characteristics	Overall (n=169)
**Gender**	
Male	110 (64.7%)
Female	59 (35.3%)
**Age**	
<60	82 (48.5%)
≥60	87 (51.5%)
**Nuclear fission**	
<5	63 (37.3%)
≥5	104 (61.5%)
Unknown	2 (1.2%)
**Pathologic subtypes, n (%)**	
Epithelioid	16 (9.5%)
Spindle	108 (63.9%)
Mixed	32 (18.9%)
NA	13 (7.7%)
**NIH grading, n (%)**	
Extremely low risk	1 (0.6%)
Low risk	18 (10.7%)
Moderate risk	23 (13.6%)
High risk	127 (75.1%)
**Tumor diameter, cm**	
<5	33 (19.5%)
≥5	136 (80.5%)
**Multifocality**	
No	99 (57.6%)

Yes	70 (41.4%)
**Combined organ resection, n (%)**	
Simple tumor resection	22 (12%)
Tumor and gastrointestinal tract resection	95 (56.2%)
Tumor, gastrointestinal tract, and extra organ resection	52 (30.8%)
**Tumor texture**	
Hard	121 (71.6%)
Soft	48 (28.4%)
**Transfusion**	
No	152 (89.9%)
Yes	17 (10.1%)
**Tumor classification**	
Primary	120 (71%)
Recurrence	49 (29%)
**Preoperative Imatinib, n (%)**	
No	128 (75.7%)
Yes	41 (24.3%)
**Mutation site, n (%)**	
Non kit-11	38 (22.5%)
kit-11	101 (59.8%)
PDGFRA	9 (5.3%)
other	6 (3.6%)
NA	15 (8.9%)
**Ki-67 index (%)**	
<15	99 (58.6%)
≥15	61 (36.1%)
NA	9 (5.3%)

**Preoperative WBC (10^9/L), Mean ± SD**	5.39 ± 2.46
**Preoperative RBC (10^9/L), Mean ± SD**	3.88 ± 0.76
**Preoperative Neutrophils (10^9/L), Mean ± SD**	3.56 ± 2.27
**Preoperative Lymphocyte (10^9/L), Mean ± SD**	1.32 ± 0.50
**Preoperative Monocyte (10^9/L), Mean ± SD**	0.61 ± 2.06
**Preoperative PLT (10^9/L), Mean ± SD**	215.4 ± 104.5

Table S4. Detailed gene signatures for each cell types.

**Cell types/signatures**	**Signature genes**
Epithelial cells	*EPCAM, TSPAN8, LGALS4, ELF3, PHGR1, CEACAM5, GPX2, GDF15, S100P, CLDN7, LCN2, SFN, S100A14, CEACAM6, PIGR, MGST1, SPINK1, SMIM22, PRSS3, C19orf33, MAL2, CKB*
Fibroblasts	*COL3A1, MFGE8, COL1A1, COL12A1, COL1A2, AEBP1, EFEMP2, C1R, COL5A2, TPM2, SULF1, RARRES2, GEM, RCN3, COL5A1, LUM, CCDC80, C1S, DKK3, ACTA2, CLEC11A, DCN, COL6A3, MMP11, SPON2, CTSK, PCOLCE, SERPINF1, ANTXR1, MXRA8, FBLN1, SFRP2, EMILIN1, THBS2*
Endothelial cells	*RAMP2, PLVAP, VWF, RAMP3, CLDN5, VWA1, ESAM, EGFL7, HYAL2, AQP1, INSR, CLEC14A, FAM167B, CD34, ACKR1, SELE, ARHGAP29, PODXL, CDH5, APLNR, CALCRL*
Stroma cells	*IGFBP7, SPARC, COL4A1, SPARCL1, MGP, COL4A2, IGFBP4, COL15A1, PTRF, PRKCDBP, CAV1, CALD1, HTRA1, COL18A1, NNMT, CYR61, FSTL1, COL6A2, CTGF, IGFBP5, MMP2, MYL9, CTHRC1, BGN, PDLIM7, TIMP3*
T cells	*TRAC, CD7, CD3E, CD2, TRBC1, FYN, CD3G, TIGIT, LCK, KLRB1, CD96, CD247, ICOS, RORA, CD3D, TRBC2*
Cytotoxic Lymphocytes	*CCL5, GZMB, NKG7, GNLY, IFNG, PRF1, GZMK, GZMH, GZMA;*
CD8+ T cells	*CD8A, CD8B*
Regulatory T cells	*TNFRSF18, IL2RA, FOXP3*

NK cells	*NCR1, KLRF1, KLRC1, SH2D1B, KIR2DL1, KIR2DL3, KIR2DL4, KIR3DL1*
Plasma B cells	*MZB1, DERL3, CD38, TNFRSF17;*
CD20+ B cells	*MS4A1, BANK1, VPREB3, TNFRSF13C, CD22, CD19*
Myeloid cells	*CCL4, SPP1, CCL3L3, IL1B, FCER1G, PTGS2, APOC1, TYROBP, MMP9, S100A9, C1QB, C1QA, CD68, CD14, IL1RN, AIF1, LST1, C1QC, FCGR2A, S100A8, SPI1, MS4A6A, FCGR3A, PLEK, MS4A7, CYBB*
12-Chemokines signature	*CCL2,CCL4,CCL5,CCL8,CCL18,CCL19,CCL21,CXCL9,CXCL10,CXCL11,CXCL13*
50-Genes signature	*FDCSP,CR2,CXCL13,LTF,CD52,MS4A1,CCL19,LINC00926,LTB,CORO1A,CD7B,TXNIP,CD19,LIMD2,CD37ARHGAP45,BLK,TMC8,CCL21,PTPN6,ATP2A3,IGH,SPIB,TMSB4X,CXCR4,NCF1,CD79A,ARHGAP9,DEF6,EVL,TBC1D10C,RASAL3,INPP5D,RNASET2,RASGRP2,TNFRSF13C,RAC2,CD22,ARHGEF1,AC103591.3,TRAF3IP3,HLA-DQB1,CD53,ARHGAP4,TRBC2,POU2AF1,TRAF5,OGA,FCRL3,HLA-DQA1*
39-Genes signature	*CCL2,CCL3,CCL4,CCL5,CCL8,CCL18,CCL21,CXCL9,CXCL10,CXCL11,CXCL13,CD200,FBLN7,ICOS,SGPP2,SH2D1A,TIGIT,PDCD1,CD4,CCR5,CXCR3,CSF2,IGSF6,IL2RA,CD38,CD40,CD5,MS4A1,SDC1,GFI1,IL1R1,IL1R2,IL10,CCL20,IRF4,TRAF6,STAT5A,TNFRSF17*

Tfh cell-Genes signature	*CXCL13,CD200,FBLN7,ICOS,SGPP2,SH2D1A,TIGIT,PDCD1,CXCR5*

Table S5. Detailed information of antibodies used for mIHC staining.

REAGENT	SOURCE	IDENTIFIER
Anti-rabbit CD3, Clone: SP7	Abcam	Cat#: ab16669, RRID:AB_443425
Anti-mouse CD20, Clone: L26	Abcam	Cat#: ab9475, RRID:AB_307267
Anti-mouse CD68, Clone: KP1	Abcam	Cat#: ab955, RRID:AB_307338
Anti-rabbit CD27, Clone: EPR8569	Abcam	Cat#: ab131254, RRID:AB_11155136
Anti-rabbit CD31, Clone: Polyclonal	Abcam	Cat#: ab28364, RRID:AB_726362
Anti-rabbit IgA, Clone: EPR5367-76	Abcam	Cat#: ab124716, RRID:AB_10976507
Anti-rabbit IgD, Clone: EPR6146	Abcam	Cat#: ab124795, RRID:AB_10974228
Anti-rabbit IgG, Clone: EPR4421	Abcam	Cat#: ab109489, RRID:AB_10863040
PE, anti-human CD11c antibody	BioLegend	Cat#: 980602, RRID:AB_2888758
PE, anti-human CD8 antibody	BioLegend	Cat#: 980902, RRID:AB_2616623
Anti-mouse Pan-Keratin, Clone: 5D3/LP34	Cell Signaling Technology	Cat#: 27178, RRID: AB_3711234
Anti-rabbit CD19, Clone: D4V4B	Cell Signaling Technology	Cat#: 86916, RRID:AB_3698884
Anti-rabbit c-Kit, Clone: D3W6Y XP	Cell Signaling Technology	Cat#: 37805, RRID:AB_2799120
Anti-rabbit Cleaved Caspase-3, Clone: 5A1E	Cell Signaling Technology	Cat#: 9664, RRID:AB_2070042

Anti-rabbit Myeloperoxidase, Clone: E1E7I XP	Cell Signaling Technology	Cat#: 14569, RRID:AB_2798516
Anti-rabbit NCAM1 (CD56), Clone: E7X9M	Cell Signaling Technology	Cat#: 99746, RRID:AB_2827532
Anti-rabbit Synaptophysin, Clone: D8F6H	Cell Signaling Technology	Cat#: 25056, RRID:AB_2924257
Anti-rat CD208, Clone: 1010E1.01	Novus	Cat#: DDX0191P-100, RRID:AB_2827532
Anti-rabbit CD11b/ITGAM, Clone: E3J2F	Cell Signaling Technology	Cat#: 48893, RRID: AB_3711236
Anti-rabbit COL1A1, Clone: E8F4L	Cell Signaling Technology	Cat#: 72026, RRID:AB_2904565

Table S6. Detailed clinical and pathological information of GIST samples in the public scRNA-seq cohort (n = 7, GSE254762).

Number	Sample	Gender	Age	Primary tumor Location	Mutation site	Lines of targeted treatment	Imatinib resistance	Mitotic index (/50 HPF)
1	MP1	Male	55	Small intestine	KIT exon 9	Progression after 3 lines	Yes	>5
2	ML1	Male	55	Small intestine	KIT exon 9	Progression after 3 lines	Yes	>5
3	MP2	Male	62	Small intestine	KIT exon 11 and exon 17	First-line	No	<5
4	MP3	Male	69	Small intestine	KIT exon 11 and exon 13	Progression after 3 lines	Yes	>50
5	PG4	Male	58	/	KIT exon 11	First-line	No	<5
6	PG5	Female	63	/	KIT exon 11	First-line	No	<5
7	PG6	Female	67	/	PDGFRA exon 18	No	Yes (Primary resistance)	<5

					(D842V)			
8	PC7	Male	66	/	KIT exon 11 and exon 18	First-line	No	<5
9	ML7	Male	66	/	KIT exon 11 and exon 18	First-line	No	<5

Table S7. Detailed information of immune scRNA-seq clusters within tumor and peritumor tissues in GIST patients (n = 8).

Number	Sample	T_cells	B_cells	NK_cells	Neutrophil	Monocyte	Macrophage	Dendritic_cells	Mast_cells	Tumor	SMC	Endothelial_cells	Epithelial_cells	Fibroblast	Total
1	Tumor	4893	2394	3906	123	162	232	256	224	4062	68	56	383	362	17121
2	Tumor	169	21	645	17	40	85	68	28	6106	64	53	68	262	7626
3	Tumor	1811	126	1671	43	26	575	304	21	4511	98	24	129	346	9685
4	Tumor	3956	2804	958	1	15	432	27	121	13	77	85	35	14	8538
5	Tumor	1434	376	1339	295	364	1362	480	52	7695	147	616	174	600	14934
6	Tumor	640	280	1030	72	97	1062	288	50	7910	48	108	146	519	12250
7	Tumor	1501	4222	493	142	149	647	129	53	398	819	1037	31	1408	11029

8	Tumor	5216	1112	3047	308	584	843	818	52	284	270	1229	162	260	14185

**Number**	**Sample**	**T_cells**	**B_cells**	**NK_cells**	**Neutrophil**	**Monocyte**	**Macrophage**	**Dendritic_cells**	**Mast_cells**	**SMC**	**Endothelial_cells**	**Epithelial_cells**	**Fibroblast**	**Total**
1	Peri-tumor	316	214	84	6	3	18	7	33	34	151	732	92	1690
2	Peri-tumor	2194	56	297	10	26	32	6	19	0	5	536	7	3188
3	Peri-tumor	2176	3453	472	802	74	83	50	45	3	311	1335	88	8892
4	Peri-tumor	3766	2305	372	16	36	244	97	142	327	2929	47	1427	11708
5	Peri-tumor	2246	200	619	195	69	246	203	1647	287	2797	190	4822	13521
6	Peri-tumor	3190	2939	410	157	40	22	12	235	55	217	881	69	8227

7	Peri-tumor	298	2549	86	5	2	20	6	13	10	97	284	19	3389
8	Peri-tumor	648	594	317	6	6	68	13	65	5	21	32	83	1858

Table S8. Top 100 DEGs information across B cell subsets.

Naive_B_cells	Memory_B_cells	GC_B_cells	IgA_PCs	IgG_PCs
IGHD	*HLA-DRA1*	*MS4A12*	*IGHA2*	*IGHGP*
HLA-DRA	*MS4A11*	*LRMP*	*IGHA1*	*IGHG3*
CXCR4	*LTB1*	*ACTB2*	*JCHAIN*	*IGHG1*
MS4A1	*BANK11*	*HLA-DRB12*	*IGHM*	*IGHG21*
CD37	*CD521*	*HLA-DQA12*	*IGHG4*	*IGLC31*
CD52	*CXCR41*	*LCP12*	*IGKC*	*IGLC21*
ZFP36L1	*HLA-DPB11*	*ACTG12*	*IGHJ4*	*MZB11*
CD22	*CD241*	*LAPTM52*	*HSP90B1*	*XBP11*
BTG1	*CD371*	*CD522*	*SSR4*	*ITM2C1*
CD69	*CD741*	*PLEK1*	*DERL3*	*PTP4A3*
LAPTM5	*NAP1L11*	*HLA-DRA2*	*SLAMF7*	*PECAM11*
HLA-DPB1	*LAPTM51*	*HLA-DPA12*	*RRBP1*	*PRDX41*
HLA-DPA1	*HLA-DPA11*	*ITGAX*	*HSPA5*	*SSR41*

CD74	*ITGB1*	*HLA-DRB52*	*XBP1*	*HSPA1A*
HLA-DRB1	*HLA-DRB11*	*HLA-DPB12*	*SEC11C*	*SDC11*
SELL	*CD82*	*HLA-DQB12*	*IGLC2*	*IGKC1*
BANK1	*ZFP36L11*	*CD742*	*MZB1*	*ADA21*
PTPRC	*TXNIP1*	*UCP22*	*ABCG1*	*DERL31*
HLA-DQB1	*RIPOR21*	*PFN12*	*IGLC3*	*FKBP21*
HLA-DMB	*RPLP21*	*CD222*	*PDIA4*	*RRBP11*
FCMR	*IRF81*	*LMO2*	*HERPUD1*	*ERLEC11*
HVCN1	*HLA-DQB11*	*FGR*	*SDC1*	*PIM21*
PAX5	*PTPRC1*	*SELL2*	*PRDM1*	*PSAP1*
IRF8	*ACTB1*	*CFL12*	*HDLBP*	*HSPA1B*
RIPOR2	*FCMR1*	*ARPC52*	*PIM2*	*SLAMF71*
ETS1	*SP1101*	*ARPC22*	*TENT5C*	*RGS12*
FAM129C	*HLA-DQA11*	*LIMD22*	*PABPC4*	*GAS61*
HLA-DQA1	*RPL111*	*GAPDH1*	*MANF*	*CD631*
RPS27	*RPL391*	*BASP12*	*DNAJB9*	*ITGA61*

REL	*RPS231*	*SH3BGRL32*	*SSR3*	*JSRP11*
TXNIP	*RPS271*	*MARCKSL1*	*ITM2C*	*TMEM591*
RASGRP2	*CYBB1*	*CORO1A2*	*FKBP2*	*DUSP51*
SMAP2	*RPS201*	*ARHGDIB2*	*SEL1L*	*CPEB41*
RPLP2	*1-Mar*	*CD532*	*CREB3L2*	*SEC11C1*
TRBC2	*BTG11*	*POU2F22*	*PRDX4*	*FKBP111*
FCRL1	*ARHGDIB1*	*FCMR2*	*NUCB2*	*SEL1L1*
SP110	*YWHAZ1*	*RFTN1*	*PDIA6*	*TENT5C1*
LTB	*LCP11*	*ACTR32*	*SELENOS*	*FBXW71*
RPS23	*RPS211*	*SMIM142*	*PGC*	*NUCB21*
BIRC3	*RAC21*	*FCRL3*	*FKBP11*	*SPAG41*
HLA-DRB5	*HLA-DMB1*	*AICDA*	*HYOU1*	*TMEM2581*
RPS21	*SMAP21*	*CD372*	*FNDC3B*	*RPN21*
RPL39	*PRKCB1*	*PTPRC2*	*CCPG1*	*CD381*
SP100	*TPM31*	*HLA-DMB2*	*RPN2*	*TRIB11*

BCL11A	*SMIM141*	*COTL11*	*TNFRSF17*	*FCRL52*
RPL11	*RPS121*	*CD192*	*CALR*	*NPC21*
PLEKHA2	*RPL321*	*ACTR22*	*TRAM1*	*JUN*
RPL32	*RPS291*	*HLA-DMA2*	*CRELD2*	*TECR1*
NAP1L1	*REL1*	*TMSB4X2*	*ELL2*	*RGCC1*
CIITA	*RPL381*	*CNN22*	*SPCS3*	*CHST2*
RCSD1	*PTMA1*	*CSK1*	*SPCS1*	*PRDM11*
RPS3A	*RPL91*	*LBH2*	*CKAP4*	*HSPB11*
ARHGDIB	*RPS71*	*PAX52*	*CLPTM1L*	*APOL6*
STK17B	*TLR10*	*CTSH1*	*SPCS2*	*H1FX1*
TRIM22	*RPL311*	*PTPN18*	*DNAJC1*	*ELL21*
RPL10A	*RPL10A1*	*EZR2*	*TMEM258*	*LMAN11*
RPS20	*SELL1*	*PARP11*	*TXNDC11*	*PDK11*
KLF2	*SWAP701*	*CAPZB2*	*UBE2J1*	*PLD31*
SWAP70	*RPS61*	*TPM32*	*SEC61B*	*CITED21*
RPL9	*STK17B1*	*H3F3A2*	*CANX*	*SELENOS1*

RPL19	*ZFAS11*	*SCIMP*	*TMED9*	*TXNDC151*
ACTB	*KIAA15511*	*RAC22*	*P4HB*	*FOS*
FOXP1	*RPL301*	*GDI21*	*ITGA6*	*FNDC3B1*
PTMA	*RPS181*	*S100A4*	*DNAJC3*	*SPCS31*
RALGPS2	*RPL191*	*SPIB*	*HM13*	*MAN1A11*
CYBB	*ACTR21*	*HMGA1*	*KDELR1*	*CCDC88A2*
RPL12	*ATP2B11*	*BMP2K*	*MYDGF*	*SPCS21*
SH3BGRL3	*RPL341*	*LBR*	*SRPRA*	*SEC14L11*
RPS27A	*RPS141*	*SYK2*	*TMEM59*	*IFI61*
RPSA	*PFN11*	*PTPN62*	*RPN1*	*SELENOK1*
RPL30	*RPS3A1*	*FCRLA2*	*ERLEC1*	
LCP1	*GAPT*	*MOB1A2*	*SELENOK*	
MTPN	*RPS27A1*	*PLEKHA21*	*DUSP5*	
PRKCB	*RASGRP21*	*IRF82*	*CD63*	
P2RX5	*RPSA1*	*CD79B2*	*SPATS2*	

RPS6	*SH3BGRL31*	*ITSN22*	*LMAN1*
RPL31	*RPS31*	*YWHAB2*	*IL6ST*
RPL34	*EEF1A11*	*DHRS9*	*MANEA*
RPS12	*TOMM71*	*PPIA2*	*PDK1*
RPS26	*HLA-DRB51*	*SWAP702*	*SDF2L1*
LYN	*HLA-DQA21*	*SEPT72*	*PSAP*
YWHAZ	*RPS131*	*RGS13*	*TXNDC15*
ACTR2	*CORO1A1*	*TMSB102*	*PPIB*
IFI16	*ETS11*	*ETS12*	*TMED10*
CORO1A	*TAGLN21*	*ANP32B2*	*ADA2*
ADD3	*TRAF51*	*MTPN2*	*GLCCI1*
RPL37	*INPP5D1*	*ALOX5AP*	*NT5DC2*
RPL22	*RPS251*	*ARPC4*	*ERN1*
RPS11	*EZR1*	*LYN2*	*SRPRB*
RPL38	*VPREB3*	*PIP4K2A*	*SLC1A4*
SEPT7	*UCP21*	*HMGN12*	*LINC02362*

ZFAS1	*COMMD61*	*PEA15*	*ACADVL*
RPS3	*RPL13A1*	*RHOA1*	*FNDC3A*
RPL13	*TRIM221*	*PXK*	*OS9*
CD24	*PABPC11*	*NAP1L12*	*GSTP1*
RPL23	*CD481*	*ITGB2*	*KDELR2*
LBH	*GPR183*	*CDC422*	*SPAG4*
TRAF5	*COTL1*	*SKAP2*	*INSR*
RPS29	*SP1001*	*PTPN12*	*SEC61G*
RPS18	*RALGPS21*	*CLEC2D*	*JSRP1*

Table S9. Univariate and multivariate analysis of overall survival (OS) in serum GIST cohort (n=169).

Variables	Univariate analysis		Multivariate analysis	
	**HR (95%CI)**	***P* value**	**HR (95%CI)**	***P* value**
Age, years (>60 vs. ≤60)	1.488 (0.643 - 3.447)	0.353		
Sex (Female vs. Male)	1.107 (0.473 - 2.592)	0.814		
Tumor size (>5cm vs. ≤5cm)	1.638 (0.479 - 5.607)	0.432		
Tumor number (Multiple vs. Single)	3.903 (1.855-8.211)	**<0.001**	1.961 (0.809-4.751)	0.136
Tumor texture (Hard vs. Soft)	2.783 (1.306-5.928)	**0.008**	0.625 (0.266-1.468)	0.281
Mitotic index (>5/50 HPF vs. ≤5/50 HPF)	4.600 (1.492 - 14.185)	**0.008**	2.169 (0.286 - 16.461)	0.454

NIH grading (High vs. Low)	4.906 (1.480-16.261)	**0.009**	2.187 (0.876 - 5.465)	0.094
WHO prognosis group (4-6 vs. 1-3)	2.355 (1.003 - 5.530)	**0.049**	2.414 (0.666 - 8.749)	0.180
Ki-67 index (>15% vs. ≤15%)	4.994 (2.354-10.594)	**<0.001**	3.212 (1.242-8.308)	**0.016**
Tumor status (Recurrent vs. Primary)	6.119 (2.330 - 16.070)	**< 0.001**	2.436 (0.531 - 11.175)	0.252
Kit 11 mutation (Yes vs. No)	0.541 (0.237 - 1.237)	0.146		
Serum IgG level (High vs. Low)	0.925 (0.879 - 0.973)	**0.003**	0.936 (0.867 - 1.011)	0.094
Serum IgA level (High vs. Low)	0.619 (0.349 - 1.096)	0.100		
